# CRISPR/Cas diagnostics for *Klebsiella pneumoniae*: from resistance and hypervirulence markers to clinically interpretable risk reporting

**DOI:** 10.3389/fbioe.2026.1949504

**Published:** 2026-09-11

**Authors:** Weijia Yi, Hongzhi Luo, Fukang Luo, Qianyi Li, Xiaoxue Huang, Qiujie Yu, Guozhen Deng, Shan Liu

**Affiliations:** 1 West China Hospital Sichuan University Jintang Hospital, Jintang First People’s Hospital, Chengdu, China; 2 Department of Laboratory Medicine, Public Health Clinical Center of Chengdu, Chengdu, China; 3 Department of Laboratory Medicine, The Third Affiliated Hospital of Zunyi Medical University (The First People’s Hospital of Zunyi), Zunyi, China; 4 The Second Affiliated Hospital of Guizhou University of Traditional Chinese Medicine, Guiyang, China; 5 Department of Neurology, Sichuan Provincial People’s Hospital, University of Electronic Science and Technology of China, Chengdu, China; 6 Genetic Diseases Key Laboratory of Sichuan Province, Department of Medical Genetics, Sichuan Academy of Medical Sciences and Sichuan Provincial People’s Hospital, University of Electronic Science and Technology of China, Chengdu, China

**Keywords:** antimicrobial resistance-associated targets, CRISPR/Cas, CRKP, hvKp, hypervirulence-associated markers, *Klebsiella pneumoniae*, risk reporting

## Abstract

*Klebsiella pneumoniae* (Kp) is a major cause of healthcare-associated and community-onset invasive infections. The emergence of carbapenem-resistant Kp (CRKP), hypervirulent Kp (hvKp), and resistance–hypervirulence convergence has increased the need for rapid tests that provide organism context and clinically interpretable risk information. CRISPR/Cas diagnostics combine programmable sequence recognition with flexible signal generation and can be integrated with isothermal amplification, portable readouts, and multiplex workflows. Available studies support the analytical feasibility of detecting Kp- or *Klebsiella pneumoniae* species complex (KpSC)-associated organism-context targets, major resistance genes, and hypervirulence-associated markers, but clinical and workflow validation remains limited because many studies use small cohorts, cultured isolates, or spiked matrices. Here, clinically interpretable risk reporting means translating validated molecular findings into bounded categories—organism context, gene-associated resistance risk, hypervirulence-associated risk, and suspected resistance–hypervirulence convergence risk—while stating interpretive limits and appropriate confirmatory actions. We critically evaluate CRISPR/Cas engineering, target selection, multiplexing, specimen-specific workflows, and validation requirements, and propose a conceptual four-layer target-to-report framework for prospective evaluation. CRISPR/Cas-based Kp testing should complement, rather than replace, culture, phenotypic antimicrobial susceptibility testing, virulence assessment, and genomic confirmation.

## 1 Introduction

*Klebsiella pneumoniae* (hereafter, Kp) is a major opportunistic pathogen in clinical microbiology and a leading cause of pneumonia, bloodstream infection, urinary tract infection, and other invasive infections, particularly in intensive care unit (ICU) settings and among immunocompromised patients (Martin and Bachman, 2018; Paczosa and Mecsas, 2016). It can cause both healthcare-associated and community-acquired infections and may progress from colonization of the respiratory, gastrointestinal, or urinary tract to invasive disease when host barriers or immune defenses are compromised (Gorrie et al., 2017; Russo and Marr, 2019). Bacterial infections and antimicrobial resistance are major public health challenges, and Kp is a leading antimicrobial-resistant Gram-negative pathogen (Antimicrobial Resistance Collaborators, 2022; GBD, 2019 Antimicrobial Resistance Collaborators, 2022). In high-risk clinical scenarios, the diagnostic challenge therefore extends beyond detecting Kp itself: early testing should also identify resistance-associated, hypervirulence-associated, or resistance–hypervirulence convergence signals that may affect treatment and infection control decisions (Lan et al., 2021; Russo et al., 2018).

This need is particularly urgent in sepsis and other severe infections, in which timely empirical antimicrobial therapy, pathogen confirmation, and infection control measures strongly influence outcomes (Evans et al., 2021). When pathogen identification and resistance information are delayed, clinicians often rely on broad-spectrum empirical therapy, which may delay targeted treatment and increase antimicrobial selection pressure (Peri et al., 2024). Kp may produce extended-spectrum β-lactamases (ESBLs), exhibit carbapenem resistance, or acquire resistance to last-line agents (Paterson and Bonomo, 2005; Pitout et al., 2015; Poirel et al., 2017; Tamma et al., 2022), and may display multidrug-resistant, extensively drug-resistant, or pandrug-resistant phenotypes (Magiorakos et al., 2012). Early molecular detection of ESBL genes, carbapenemase genes, mobile colistin-resistance markers, or *tet(X)*-associated tigecycline-resistance determinants may support timely antimicrobial review, isolation precautions, infection prevention measures, and outbreak assessment, pending phenotypic and epidemiological confirmation (Paterson and Bonomo, 2005; Pitout et al., 2015; Poirel et al., 2017; Tamma et al., 2022; Banerjee and Humphries, 2017; He et al., 2019).

Hypervirulent Kp (hvKp) is commonly associated with invasive community-acquired infections, pyogenic liver abscess, and metastatic infection (Russo and Marr, 2019). At the molecular level, *rmpA/rmpA2*, siderophore-associated loci, and *peg-344* are commonly used as indicators of hypervirulence-associated risk (Russo et al., 2018). However, no single virulence gene independently defines hvKp. Likewise, K1/K2 capsular types, ST23, or a positive string test should not automatically be equated with a hypervirulent phenotype (Russo et al., 2024; Shi et al., 2018). In particular, *magA* should be interpreted as a K1-associated capsular marker rather than as a universal K1/K2 marker (Struve et al., 2005). A particularly concerning scenario is the convergence of antimicrobial resistance and hypervirulence. Fatal outbreaks involving ST11 carbapenem-resistant hypervirulent Kp illustrate how a high-risk clonal background combined with resistance and virulence elements can complicate treatment, infection control, and public health surveillance (Gu et al., 2018).

Culture-based identification and antimicrobial susceptibility testing (AST) remain foundational components of clinical microbiology (Gajic et al., 2022). They enable recovery of viable isolates, phenotypic susceptibility testing, strain preservation, and whole-genome sequencing (WGS)-based source tracing, but the complete culture-to-identification/AST pathway is slower than direct molecular screening and varies with specimen type and laboratory workflow (Gajic et al., 2022; Calderaro and Chezzi, 2024). Matrix-assisted laser desorption/ionization time-of-flight mass spectrometry (MALDI-TOF MS) shortens isolate-identification time but still requires prior culture (Calderaro and Chezzi, 2024). PCR and quantitative PCR (qPCR) are mature, sensitive, and relatively standardized and can detect Kp-associated targets and common resistance or virulence genes (Compain et al., 2014). However, most PCR/qPCR assays remain single-target or limited-panel tests, and their results require phenotypic and epidemiological context (Yang and Rothman, 2004). WGS can resolve clonal and capsular backgrounds and support outbreak source tracing (Zhou et al., 2016; Wyres et al., 2016; Snitkin et al., 2012). Plasmid-resolved genomic analysis can further clarify the genetic context of carbapenemase and suspected convergence signals (David et al., 2020), whereas metagenomic next-generation sequencing (mNGS) can support complex-sample and unknown-pathogen identification (Simner et al., 2018). Nevertheless, cost, turnaround time, and bioinformatic interpretation requirements limit routine use of genome-scale approaches for early bedside decision-making (Simner et al., 2018).

In this context, CRISPR/Cas diagnostics are best viewed as a complementary intermediate layer between conventional rapid molecular testing and definitive microbiological or genomic characterization. Their distinguishing features are programmable sequence recognition, compatibility with low-temperature or isothermal amplification, flexible fluorescence/lateral-flow/electrochemical readouts, and the capacity to combine organism-context, resistance-associated, and hypervirulence-associated targets within compact workflows (Chen et al., 2018; Gootenberg et al., 2017; Kaminski et al., 2021; Liu et al., 2025; Liborio et al., 2024). These attributes may shorten the time to a bounded molecular risk report in selected settings, particularly when the result is explicitly linked to downstream AST, isolate preservation, infection control assessment, or WGS/mNGS confirmation. CRISPR/Cas should therefore not be positioned as a replacement for culture, qPCR, MALDI-TOF MS, or WGS, but as a potentially useful screening and triage layer whose value depends on complete specimen-to-report performance and clinically interpretable output.

For Kp diagnostics, the clinically relevant question extends beyond pathogen detection to early interpretation of antimicrobial resistance-associated risk, hypervirulence-associated risk, and possible resistance–hypervirulence convergence. Species-level detection alone is usually insufficient to guide antimicrobial treatment in severe infections or in clinical scenarios with a high risk of antimicrobial resistance. Likewise, a resistance gene does not define a complete resistance phenotype, and a single virulence marker does not establish hvKp. A clinically useful report should therefore state whether a validated Kp-associated or *K. pneumoniae* species complex (KpSC)-associated target was detected, list the covered resistance- and hypervirulence-associated markers, and indicate the relevant interpretive boundary and confirmatory pathway. In mixed or non-isolate specimens, concurrent detection of organism-context, resistance, and hypervirulence-associated targets is reported as specimen-level co-detection and a suspected convergence risk signal; it does not establish same-strain carriage or colocalization on the same genetic vehicle.

Compared with a recent Kp-focused CRISPR/Cas review that emphasized diagnostic and therapeutic applications (Feng and Yin, 2026), this review places Kp testing within a continuous clinical decision pathway. This pathway includes organism-context detection, antimicrobial resistance risk, hypervirulence-associated risk, possible resistance–hypervirulence convergence, and subsequent AST/WGS confirmation. Rather than ranking platforms solely by their lowest reported limits of detection, we assess whether their targets and outputs can support interpretable reports and predefined confirmatory workflows.

**FIGURE 1:**
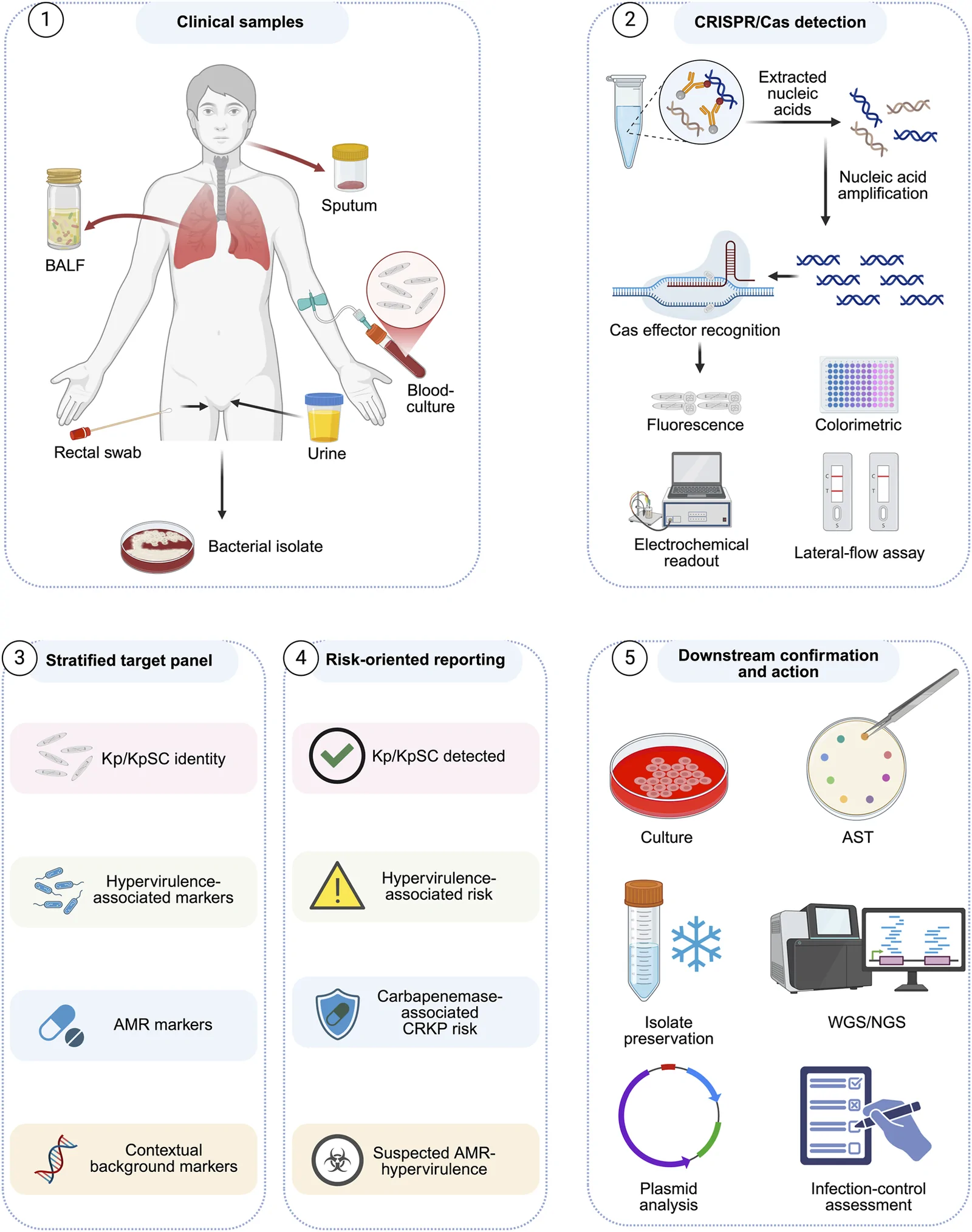
Conceptual workflow for CRISPR/Cas-based risk reporting in *Klebsiella pneumoniae*. AMR, antimicrobial resistance; AST, antimicrobial susceptibility testing; BALF, bronchoalveolar lavage fluid; CRKP, carbapenem-resistant *K. pneumoniae*; KpSC, *K. pneumoniae* species complex; NGS, next-generation sequencing; WGS, whole-genome sequencing.

This review first summarizes the applicability of CRISPR/Cas effectors, amplification strategies, and readout methods in Kp detection. It then organizes the available evidence across four dimensions: species identification, antimicrobial resistance detection, hypervirulence-associated markers, and multiplex reporting. On this foundation, we propose a scenario-driven stratified target panel and a conceptual report-triggering framework. Finally, clinical translation pathways, validation standards, and future directions are discussed. The proposed sample-to-report and downstream-confirmation workflow is summarized in Figure 1.

### 1.1 Review scope and literature search strategy

This article is a structured narrative review supported by a study-level evidence inventory rather than a formal systematic review or meta-analysis. PubMed/PMC was the principal bibliographic source, supplemented by publisher-platform and Chinese-language searches. No lower publication-date limit was applied; searches covered records available from database inception through 6 July 2026 and combined terms related to Kp/KpSC/CRKP, CRISPR/Cas systems, diagnosis, antimicrobial resistance, hypervirulence, multiplex detection, point-of-care testing, specimen-specific workflows, and rapid antimicrobial susceptibility testing. Complete source-specific strategies and update dates are provided in Supplementary Table S1.

Records were deduplicated primarily using DOI/PMID identifiers and screened by title and abstract, followed by full-text assessment against predefined eligibility criteria. We included original research and technical or methodological studies relevant to Kp/KpSC detection, resistance- or hypervirulence-associated targets, multiplex diagnostics, rapid phenotypic AST, or directly transferable diagnostic engineering; reviews, therapeutic or gene-editing studies, endogenous CRISPR-Cas epidemiology, non-CRISPR diagnostic studies, and insufficiently relevant reports were excluded. After eligibility assessment, 53 core or closely related original studies were retained. Of these, 38 directly addressed Kp/KpSC-focused diagnostic, resistance, hypervirulence, or rapid-AST applications and are summarized in Supplementary Table S2; the remaining retained studies provided transferable CRISPR engineering or validation evidence discussed in the main text. Quantitative values were verified against the original articles and retained in their reported units and experimental contexts. Because of substantial methodological heterogeneity, evidence was synthesized qualitatively. Kp-focused study-level evidence is summarized in Supplementary Table S2.

## 2 Technical basis and platform engineering of rapid CRISPR/Cas detection

### 2.1 Cas effectors and signal transduction mechanisms

CRISPR/Cas diagnostics use guide RNA (gRNA) or CRISPR RNA (crRNA) to achieve sequence-specific recognition and convert the presence of target nucleic acids into detectable signals (Kaminski et al., 2021). A complete CRISPR/Cas biosensor typically comprises sample processing, target amplification, Cas-mediated recognition, and endpoint readout modules (Yin et al., 2024). Its value lies not only in analytical sensitivity but also in its ability to integrate sequence recognition, enzymatic signal generation, and engineered readout within a programmable sensing system (Verosloff et al., 2022). In Kp diagnostics, these modules are clinically relevant only when linked to meaningful target combinations and bounded reporting outputs.

Cas12a and Cas12b are the most widely studied effectors in Kp-focused CRISPR diagnostics for DNA targets, resistance genes, and virulence-associated markers (Wang et al., 2025). After recognizing a double-stranded DNA (dsDNA) target adjacent to a compatible protospacer-adjacent motif (PAM), Cas12a undergoes a conformational change, activates single-stranded DNA (ssDNA) trans-cleavage, and cleaves reporter probes (Chen et al., 2018; Swarts and Jinek, 2019). This mechanism facilitates coupling with products generated by isothermal amplification or PCR. Cas12b is also a DNA-targeting effector, and some variants function at elevated temperatures, enabling integration with loop-mediated isothermal amplification (LAMP) or multiple cross-displacement amplification (MCDA) (Li et al., 2019; Nguyen et al., 2023). For example, CRISPR-KP introduces a Cas12b-recognizable PAM through engineered MCDA primers, enabling one-pot MCDA–Cas12b detection (Zhou et al., 2025).

Cas13a recognizes RNA rather than DNA, and its collateral-cleavage substrate is a single-stranded RNA (ssRNA) reporter. The original SHERLOCK platform demonstrated that Cas13a combined with recombinase polymerase amplification (RPA) and T7 transcription enabled highly sensitive nucleic acid detection and multiplex typing. Detection of Kp genomic DNA and resistance genes with Cas13 generally requires a transcription step, making the workflow less direct than Cas12-based DNA detection (Gootenberg et al., 2017; Cao et al., 2024). Assays targeting rRNA, mRNA, or other RNA signals may serve as proxies for bacterial activity only after the relationships among RNA persistence, organism viability, and specimen type have been validated (Xue et al., 2022). Cas13a may therefore be particularly useful for RNA-target detection (Xue et al., 2022), lyophilized paper-based testing (Bhattacharjee et al., 2024), or multiplex-platform expansion (Thakku et al., 2022) rather than as a routine substitute for Cas12a or Cas12b in DNA-target detection.

Beyond Cas12 and Cas13, systems based on Cas9/dCas9, CasΦ, or Cas14 may provide complementary routes for target enrichment, material-based sensing, amplification-free detection, or miniaturized nucleic acid recognition (Harrington et al., 2018; Pausch et al., 2020; Xu et al., 2020). However, direct clinical evidence for Kp or resistance gene detection remains limited. These systems should therefore be regarded as transferable engineering options or future platform components rather than as core elements of current Kp risk stratification panels. Overall, the Kp-specific evidence base remains concentrated on Cas12a and Cas12b, whereas other effectors are better viewed as complementary approaches for selected specimen types or future platform development (Aman et al., 2020; Pausch et al., 2021).

### 2.2 Amplification, amplification-free detection, and control of reaction timing

**FIGURE 2:**
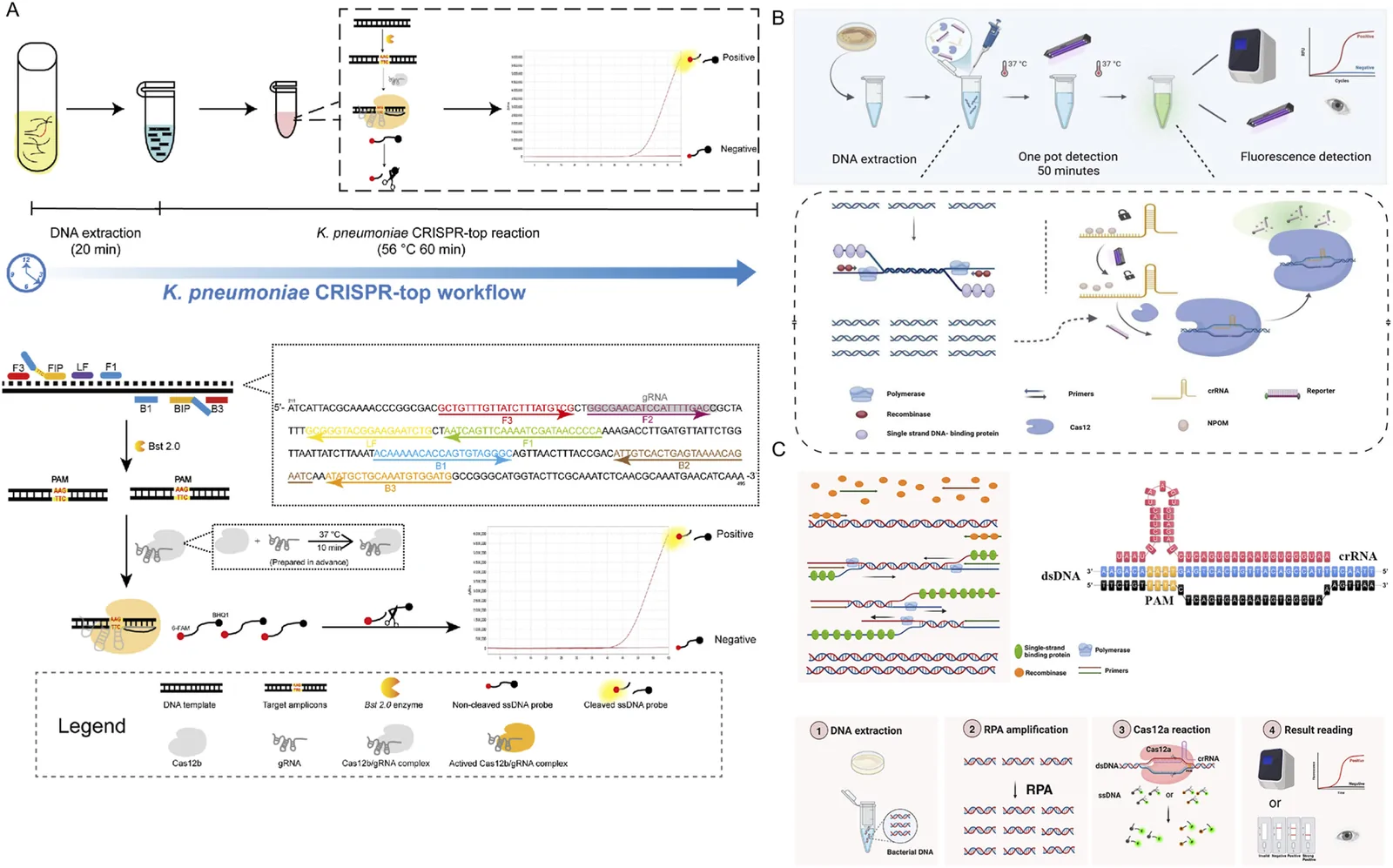
Representative CRISPR/Cas-based workflows for *Klebsiella pneumoniae* identification. **(A)** The *K. pneumoniae* CRISPR-top assay integrates LAMP amplification and Cas12b detection in a one-pot reaction after DNA extraction. Adapted from Qiu et al. (Qiu et al., 2022). Copyright © 2022 Qiu et al.; distributed under the terms of the Creative Commons Attribution 4.0 International License (CC BY 4.0). **(B)** Light-controlled one-pot RPA–CRISPR/Cas12a uses NPOM-modified caged crRNA to suppress Cas12a activity during RPA. Adapted from Pan et al. (Pan et al., 2025). Copyright © 2025 Pan et al.; distributed under the terms of the Creative Commons Attribution License (CC BY). **(C)** A two-step RPA–CRISPR/Cas12a platform amplifies the *rcsA* target and then uses Cas12a collateral cleavage for fluorescence or lateral-flow readout. Adapted from Tan et al. (Tan et al., 2024b). Copyright © 2024 Tan et al.; distributed under the terms of the Creative Commons Attribution License (CC BY).

Most CRISPR/Cas assays require target amplification to achieve clinically relevant sensitivity (Kaminski et al., 2021). RPA is typically performed at 37 °C–42 °C, requires minimal instrumentation, offers short reaction times, and has been widely applied to Kp and resistance gene detection (Lobato and O’Sullivan, 2018; Tan et al., 2024a). Recombinase-aided amplification (RAA) is similar to RPA and is suitable for one-pot and microfluidic platforms (Xing et al., 2023). LAMP has high amplification efficiency, although its complex primer design and relatively high reaction temperature may limit compatibility with some Cas systems (Becherer et al., 2020). A representative Kp LAMP–Cas12b one-pot workflow is shown in Figure 2A. MCDA uses multiple primers and has been applied to rapid Kp amplification and detection (Niu et al., 2018). In CRISPR-KP, engineered MCDA primers were specifically used to introduce a Cas12b-recognizable PAM and enable one-pot Cas12b detection (Zhou et al., 2025).

The principal challenges in amplification-assisted CRISPR assays are contamination control and reaction timing. Two-step methods require opening the tube to transfer amplification products, increasing the risk of aerosol carryover (Fu et al., 2025). One-pot methods reduce this risk, but premature Cas activation may cleave primers, amplification intermediates, or reporters and thereby reduce amplification efficiency. Several engineering strategies have been developed to address this temporal competition (Hu et al., 2022; Lu et al., 2022). EXORCA limits early Cas12a activity by using a suboptimal PAM, reducing competition between RPA and Cas12a (Fu et al., 2025; Lu et al., 2022). In a light-controlled one-pot RPA–Cas12a assay, ultraviolet irradiation activates Cas12a through a caged crRNA after amplification has progressed, enabling temporal control within a closed system (Hu et al., 2022; Pan et al., 2025) (Figure 2B). The suboptimal-PAM one-pot RCCS similarly showed that canonical PAMs generate stronger signals in two-step assays but may activate Cas12a prematurely in one-pot reactions, whereas suboptimal PAMs can improve the limit of detection under one-pot conditions (Lu et al., 2022).

Studies of crRNA-scaffold remodeling have provided a mechanistic basis for controlling the timing of Cas activation. Cas12a activity can be reversibly inhibited by blocking the secondary structure of the crRNA scaffold and then restored through single-strand or cooperative strand displacement (Oesinghaus and Simmel, 2019; Wang et al., 2026). This approach suggests that future one-pot Kp assays should optimize not only primers and crRNAs but also the timing of Cas activation. Low background and high sensitivity are most likely to be achieved when collateral cleavage begins only after sufficient accumulation of amplification products.

Target-amplification-free detection represents a different engineering trade-off and should not be regarded as an inherently superior approach. By avoiding pre-amplification, such methods may reduce amplification-related carryover and simplify selected workflows, but their performance depends strongly on native target abundance, host and microbial background nucleic acids, matrix interference, signal-amplification architecture, dynamic range, threshold setting, and reagent or nanomaterial reproducibility. The TCC CasΦ platform uses a collateral-cleavage cascade for low-concentration pathogen detection in serum (Chen et al., 2025), whereas the CNR platform exploits high-copy rRNA and nanocatalytic signal amplification in sterile body fluids (Xiao et al., 2025). These studies provide transferable engineering evidence rather than direct validation of Kp-specific clinical assays. Low LODs observed in one analytical system or matrix should therefore not be generalized to other specimen types without matrix-specific evaluation of specificity, reproducibility, and complete specimen-to-report performance.

### 2.3 Signal readout methods and application-specific selection

Readout selection should be matched to the intended testing scenario rather than judged solely by analytical sensitivity. Fluorescence readout is sensitive and supports real-time kinetic monitoring, making it suitable for central laboratories and assay optimization, but it requires a fluorescence reader or qPCR instrument. Lateral-flow strips permit visual interpretation in primary-care or bedside settings, although band intensity is subjective and quantitative capability is limited (Kaminski et al., 2021; Kellner et al., 2019; Posthuma-Trumpie et al., 2009). Colorimetric readout can further reduce equipment requirements; for example, the CNR method uses CuO-nanocatalyst release and 3,3′,5,5′-tetramethylbenzidine (TMB) color development for amplification-free pathogen detection (Xiao et al., 2025). Surface-enhanced Raman scattering (SERS) offers high analytical sensitivity and potential multiplex spectral encoding, and dCas9-SERS studies have shown that CRISPR-guided target capture can be coupled to amplification-free detection of multidrug-resistant bacterial targets (Kim et al., 2020).

Non-optical and device-integrated readouts further expand the range of engineering options for CRISPR diagnostics. Electrochemical platforms can be integrated with wireless modules and smartphones to support portable digital monitoring (Wu et al., 2023). Wireless dCas9–polymer-dot electrochemical sensors, for example, convert sgRNA-guided target binding after RT-LAMP into changes in electrical resistance and have been used for smartphone-assisted detection of multidrug resistance-associated markers such as *blaKPC-2* and *mecA* in samples from patients with bacterial pneumonia (Im et al., 2024). Aptamer–DNA-circuit–Cas12a systems extend signal transduction beyond purified nucleic acids by converting whole-cell recognition of resistant bacteria, including KPC-2-producing Kp, into Cas12a-mediated reporter activation (Liu et al., 2026). These examples provide transferable engineering evidence; their Kp-specific translational value depends on device reproducibility, surface chemistry, aptamer specificity, sample pretreatment, and validation in authentic clinical or field specimens.

Different readout methods are suited to different deployment scenarios. Central-laboratory workflows prioritize automation and quantitative capability; bedside or field screening favors minimal equipment and visual readouts; and networked surveillance requires digital readout and data-transmission capability. Evaluation of translational CRISPR/Cas platforms should therefore consider not only analytical sensitivity but also the intended-use scenario, sample matrix, and requirements for result interpretation.

Given these technological differences, platform comparison should not focus only on which Cas effector appears most sensitive or which readout appears fastest. The translationally relevant questions are whether the architecture can process the intended specimen reliably, support clinically meaningful target combinations, control contamination and invalid results, and generate a bounded output that is linked to an appropriate confirmatory pathway. For this reason, the following evidence sections evaluate organism-context detection, resistance-associated targets, hypervirulence-associated markers, and multiplex reporting together with specimen type, validation maturity, and downstream actionability. The principal engineering trade-offs and their translational implications are summarized in Table 1.

**TABLE 1:** Engineering options for CRISPR/Cas-based Kp testing and their translational implications.

Module or route	Principal advantages	Key limitations	Potential role and representative evidence
Cas12a	Most extensive Kp-focused evidence base among studied effectors; robust trans-cleavage; compatible with PCR, RPA/RAA, and LAMP products	PAM dependence; premature activation can suppress one-pot amplification; performance remains target-dependent	Core Kp/KpSC, resistance gene, and hypervirulence-marker testing in laboratory or portable formats (Chen et al., 2018; Swarts and Jinek, 2019; Tan et al., 2024a; Fu et al., 2025; Curti et al., 2020; Tan et al., 2024b)
Cas12b	Thermostable variants support higher-temperature one-pot reactions and integration with LAMP/MCDA.	Fewer Kp clinical studies; primer/PAM engineering and temperature matching increase assay-development burden	Closed-tube species detection and other high-temperature isothermal workflows (Li et al., 2019; Nguyen et al., 2023; Zhou et al., 2025; Qiu et al., 2022)
Cas13a	RNA recognition, strong sequence discrimination, paper/LFA compatibility, and demonstrated multiplex scalability	DNA targets require amplification plus transcription; workflow is less direct than Cas12-based DNA detection	RNA-targeted assays, paper tests, and multiplex expansion (Gootenberg et al., 2017; Cao et al., 2024; Bhattacharjee et al., 2024; Thakku et al., 2022; Liang et al., 2023)
CasΦ, Cas14, and dCas9-based routes	Compact effectors or binding-based capture strategies enable alternative signal cascades, electrochemical sensing, and target enrichment	Direct Kp clinical evidence remains limited; most examples provide transferable engineering evidence, and device or material reproducibility remains a major translational variable	Future amplification-free, SERS, electrochemical, or miniaturized platforms (Harrington et al., 2018; Pausch et al.; Xu et al., 2020; Aman et al., 2020; Pausch et al., 2021; Chen et al.; Kim et al., 2020; Im et al., 2024)
RPA/RAA amplification	Low-temperature, rapid, and compatible with simple heaters, closed-tube reactions, and microfluidics	Carryover contamination in two-step formats; primer/crRNA performance must be optimized for every target.	POCT respiratory testing, rectal screening, and focused resistance panels (Lobato and O’Sullivan, 2018; Tan et al., 2024a; Xing et al., 2023; Fu et al., 2025; Xiang et al., 2024; Hyeon et al., 2026; Tan et al., 2024b)
LAMP/MCDA amplification	High amplification efficiency; Cas12b can be matched to elevated reaction temperatures; MCDA can introduce a PAM.	Complex primer design; nonspecific amplification or cross-reactivity may complicate interpretation	One-pot species detection and visual carbapenemase screening (Zhou et al., 2025; Becherer et al., 2020; Niu et al., 2018; Qiu et al., 2022; Xu et al., 2022)
One-pot timing control	Reduces tube opening and aerosol contamination and can simplify hands-on workflow	Early Cas activation may cleave primers or intermediates and lower sensitivity	Suboptimal-PAM, light-controlled, caged-crRNA, or scaffold-switching designs (Fu et al., 2025; Hu et al., 2022; Lu et al., 2022; Pan et al., 2025; Oesinghaus and Simmel, 2019; Wang et al., 2026; Hyeon et al., 2026)
Amplification-free signal cascades	Avoid amplification carryover and may simplify workflows for high-copy targets	Dependence on nanomaterials/cascade kinetics; thresholds, linear range, and lot stability require rigorous validation	Research-stage low-load serum or sterile-body-fluid detection (Chen et al.; Xiao et al., 2025); amplification-free resistance-gene detection (Pablo-Marcos et al., 2025)
Fluorescence readout	Sensitive; supports kinetic monitoring and semiquantitative assay optimization	Requires a reader and validated positivity thresholds; not inherently sample-to-answer	Central laboratories and development-stage clinical validation (Kaminski et al., 2021; Kellner et al., 2019)
LFA or colorimetric readout	Requires minimal instrumentation and permits intuitive endpoint interpretation	Band intensity can be subjective; quantitative range is limited, and matrix interference may affect visual interpretation	Candidate bedside, primary-care, field, and infection control screening applications (Xiao et al., 2025; Kellner et al., 2019; Posthuma-Trumpie et al., 2009; Xu et al., 2022; Huang et al., 2026)
Electrochemical, SERS, and smartphone-integrated readouts	Digital capture, connectivity, potential multiplex encoding, and networked surveillance	Surface chemistry, device-to-device variation, software thresholds, and data security become part of validation	Connected POCT and portable resistance/virulence monitoring (Kim et al., 2020; Wu et al., 2023; Im et al., 2024; Liu et al., 2026)

## 3 Existing evidence for CRISPR/Cas-based species identification of *Klebsiella pneumoniae*

### 3.1 Detection of Kp-associated targets such as *rcsA*, *khe*, and *rpoB*: from analytical feasibility to clinical-specimen evaluation

Organism-context detection is among the best-studied applications of CRISPR/Cas diagnostics for Kp. However, target design must distinguish *K. pneumoniae* sensu stricto from the *K. pneumoniae* species complex (KpSC). When candidate targets have not been systematically tested for cross-reactivity with closely related species such as *Klebsiella variicola* and *Klebsiella quasipneumoniae*, reports should avoid an unqualified claim of “*K. pneumoniae* confirmed” and instead state “KpSC-associated target detected” (Wang et al., 2025; McAndrew et al., 2025; Long et al., 2017).

Closed-tube species-detection studies illustrate how a second CRISPR recognition step can improve analytical specificity while reducing open-tube handling. CRISPR-top combines LAMP with Cas12b in a single tube and, in the reported validation, showed 100% inclusivity (64/64), 100% exclusivity (41/41), 96% sensitivity, and 100% specificity relative to culture in 58 sputum specimens (Qiu et al., 2022). Its approximately 1-pg genomic DNA limit of detection (LOD) is an analytical value and should not be interpreted independently of specimen burden or extraction efficiency. RPA–Cas12a workflows provide lower-temperature alternatives and have been evaluated using respiratory specimens, but their clinical cohorts remain small. A representative two-step RPA–Cas12a workflow with fluorescence or lateral-flow readout is shown in Figure 2C. In the Wu et al. workflow, the analytical reaction comprised a 15-min RPA step followed by approximately 15 min of Cas12a detection; when the reported sputum-release step was included, fluorescence and lateral-flow results were obtained after approximately 40 and 43 min, respectively, in the study workflow (Wu et al., 2025).

A second engineering theme is minimization of preprocessing and temporal competition between amplification and Cas activity. EXORCA combined rapid lysis with a suboptimal-PAM one-pot strategy, achieved an LOD of 10 colony-forming units (CFU)/μL in cultured Kp, and was evaluated in 20 clinical samples (Fu et al., 2025). Light-controlled and caged-crRNA approaches provide an alternative means of delaying Cas activation in closed reactions (Pan et al., 2025). These strategies reduce handling and may improve one-pot compatibility, but their clinical value depends on whether simplified preprocessing remains robust across authentic specimens and whether total specimen-to-report time is reduced.

Coupling Cas12b with MCDA provides another route for species-associated detection. CRISPR-KP uses AapCas12b and engineered MCDA primers in a single-tube 59 °C reaction; the reported one-pot reaction time was approximately 40 min after DNA preparation rather than a complete specimen-to-report turnaround time. The assay achieved an LOD of 10 fg per reaction (approximately 1.6 copies) and was evaluated in 83 bronchoalveolar lavage fluid (BALF) samples (Zhou et al., 2025). This reaction-level copy estimate should not be interpreted as a clinically relevant BALF burden or converted to a CFU/mL threshold unless specimen recovery, extraction/elution, and reaction-input parameters are fully specified and validated. Because discordant results were not independently adjudicated and nucleic acid extraction and fluorescence readout were still required, the study supports analytical and workflow feasibility rather than superior clinical accuracy. Across these species-detection platforms, matrix-specific preprocessing, Kp/KpSC inclusivity/exclusivity, and complete workflow validation remain more important than the lowest reported reaction-level LOD.

### 3.2 Detection of Kp in the context of multipathogen and mixed infections

**FIGURE 3:**
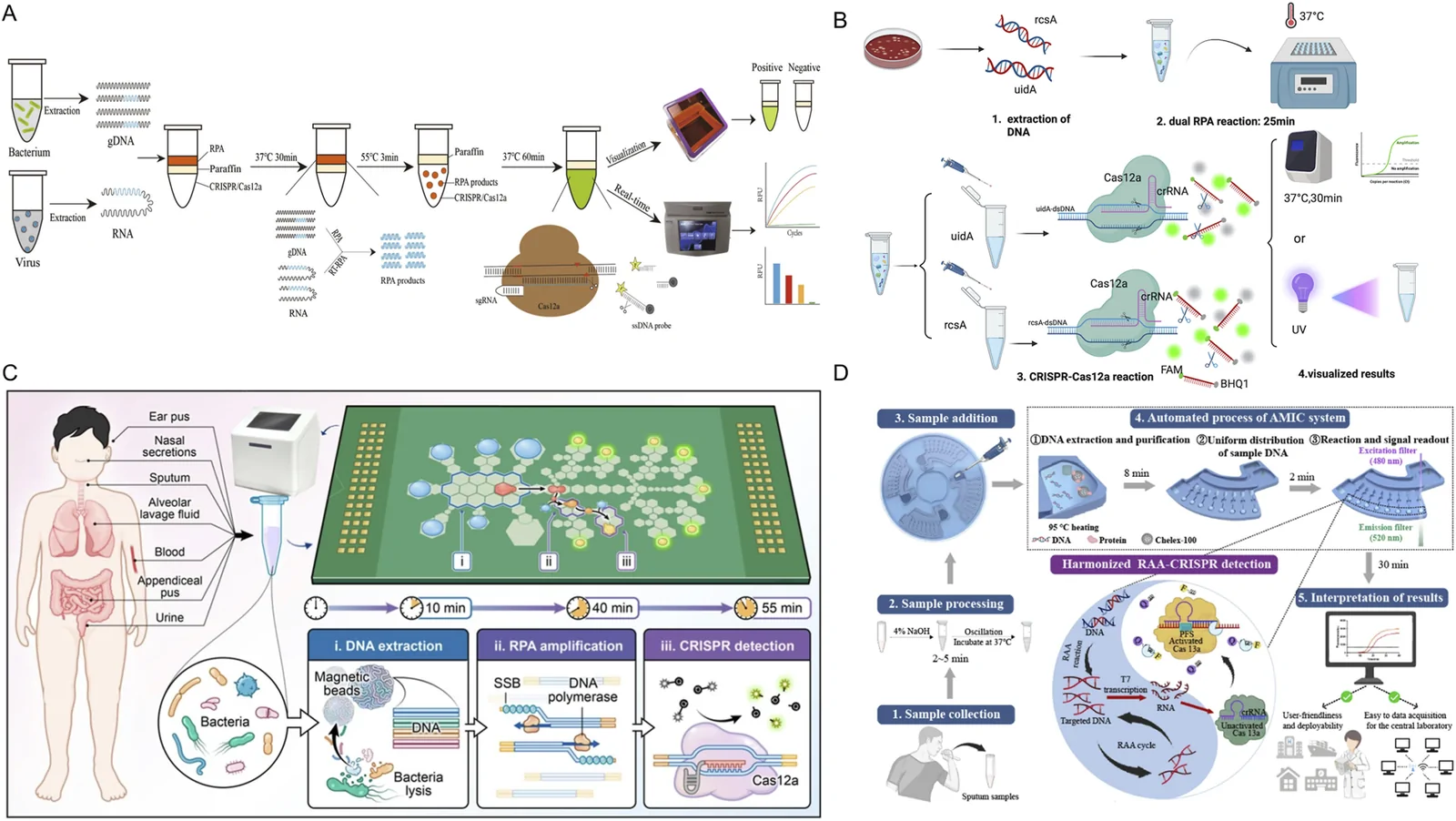
Representative multiplex and automated CRISPR-based platforms for multipathogen detection. **(A)** A paraffin-partitioned one-pot RPA–CRISPR/Cas12a workflow physically separates amplification from Cas12a detection during the initial incubation. Reproduced with permission from Tan et al. (Ta et al., 2024). Copyright © 2024 Wiley Periodicals LLC. **(B)** A dual RPA–CRISPR/Cas12a workflow co-amplifies *Escherichia coli uidA* and *Klebsiella pneumoniae rcsA* in the same RPA reaction. Adapted from Wei et al. (Wei et al., 2026). Copyright © 2026 Wei et al.; distributed under the terms of the Creative Commons Attribution 4.0 International License (CC BY 4.0). **(C)** MiND-DMF integrates magnetic-bead DNA extraction, RPA, and Cas12a detection in programmable digital-microfluidic droplets for multiplex identification of six bacterial species from multiple clinical specimen types. Adapted with permission from Xie et al. (Xie et al., 2025). Copyright 2025 American Chemical Society. **(D)** The automatic microfluidic harmonized RAA–CRISPR (AMIC) system combines sputum processing, on-chip nucleic acid extraction and distribution, harmonized RAA–Cas13a reactions, fluorescence signal readout, and centralized result interpretation for a respiratory panel targeting 12 bacterial species. Adapted with permission from Xiang et al. (Xiang et al., 2024). Copyright 2024 American Chemical Society.

Multipathogen CRISPR/Cas workflows fall broadly into two categories: broad respiratory panels and more focused bacterial combinations. Broad panels such as paraffin-partitioned one-pot RPA–Cas12a (Figure 3A) and the AMIC RAA–Cas13a system (Figure 3D) demonstrate that Kp detection can be interpreted within a broader respiratory-pathogen context rather than as an isolated positive/negative result (Ta et al., 2024; Xiang et al., 2024). AMIC integrates on-chip extraction, harmonized amplification, and fluorescence readout for a respiratory panel targeting 12 bacterial species and was evaluated in 60 clinical samples. Such platforms increase etiological breadth but do not by themselves provide resistance or hypervirulence information.

More automated multiplex systems illustrate a different translational trade-off. MiND-DMF (Figure 3C) integrates on-chip DNA extraction, RPA, and Cas12a detection in programmable digital-microfluidic droplets and completes the reported sample-to-answer workflow in approximately 55 min. The platform achieved an LOD of 100 CFU/mL for each of six bacterial targets. In 50 clinical samples from multiple specimen sources, 48 results were concordant with qPCR; subsequent 16S rRNA Sanger sequencing supported the two MiND-DMF-positive/qPCR-negative results as true positives. The study reported 100% sensitivity and target-specific specificities ranging from 98% to 100% (Xie et al., 2025). By contrast, the focused dual RPA–Cas12a workflow for *Escherichia coli uidA* and Kp *rcsA* (Figure 3B) was evaluated mainly with cultured isolates; in 30 *E. coli* isolates, 30 Kp isolates, and 20 non-target isolates, target calls were concordant with PCR (Wei et al., 2026). Together, these studies show that multiplex capacity should be judged by intended specimen, target combinations, interference testing, and reportability rather than by target count alone.

### 3.3 Common limitations of species identification studies

The studies summarized above demonstrate the analytical feasibility of CRISPR/Cas detection for Kp, but several limitations are shared across the evidence base. Most studies treat Kp detection as a single-pathogen question and do not link organism context to resistance-associated or hypervirulence-associated risk. Validation remains concentrated in respiratory specimens, cultured isolates, or selected cohorts, whereas direct blood, urine, sterile body fluids, and positive blood culture bottles are less well studied. In addition, systematic exclusion of closely related KpSC species remains incomplete for several target regions.

Reported LODs should not be ranked directly unless matrix, preprocessing, input amount, extraction/elution scheme, amplification chemistry, reaction volume, and reporting unit are comparable. Copies/reaction, copies/μL, genome equivalents, DNA mass, CFU/mL, and CFU/reaction represent different analytical bases. Extraction volume, elution volume, specimen fraction entering the reaction, and amplification efficiency can materially change the apparent LOD and the fraction of the original specimen that is interrogated. Accordingly, quantitative values in this review are retained in the units and experimental context reported by each primary study, and amplification time, Cas-reaction time, and complete sample-to-report time are distinguished whenever the source article reports these components separately. Target specificity also requires genome-wide assessment using large, geographically diverse, version-controlled genome collections to evaluate conservation, SNP/indel variation within protospacer and PAM regions, and off-target homology across KpSC, other Enterobacterales, homologous resistance gene families, and relevant background sequences. In silico assessment should be paired with wet-lab inclusivity/exclusivity testing in authentic matrices. Supplementary Table S2 provides the corresponding study-level evidence inventory.

## 4 Evidence for CRISPR/Cas detection of antimicrobial resistance- and hypervirulence-associated markers in Kp

### 4.1 Carbapenemase gene detection: from single-target confirmation to multi-target resistance-risk screening

**FIGURE 4:**
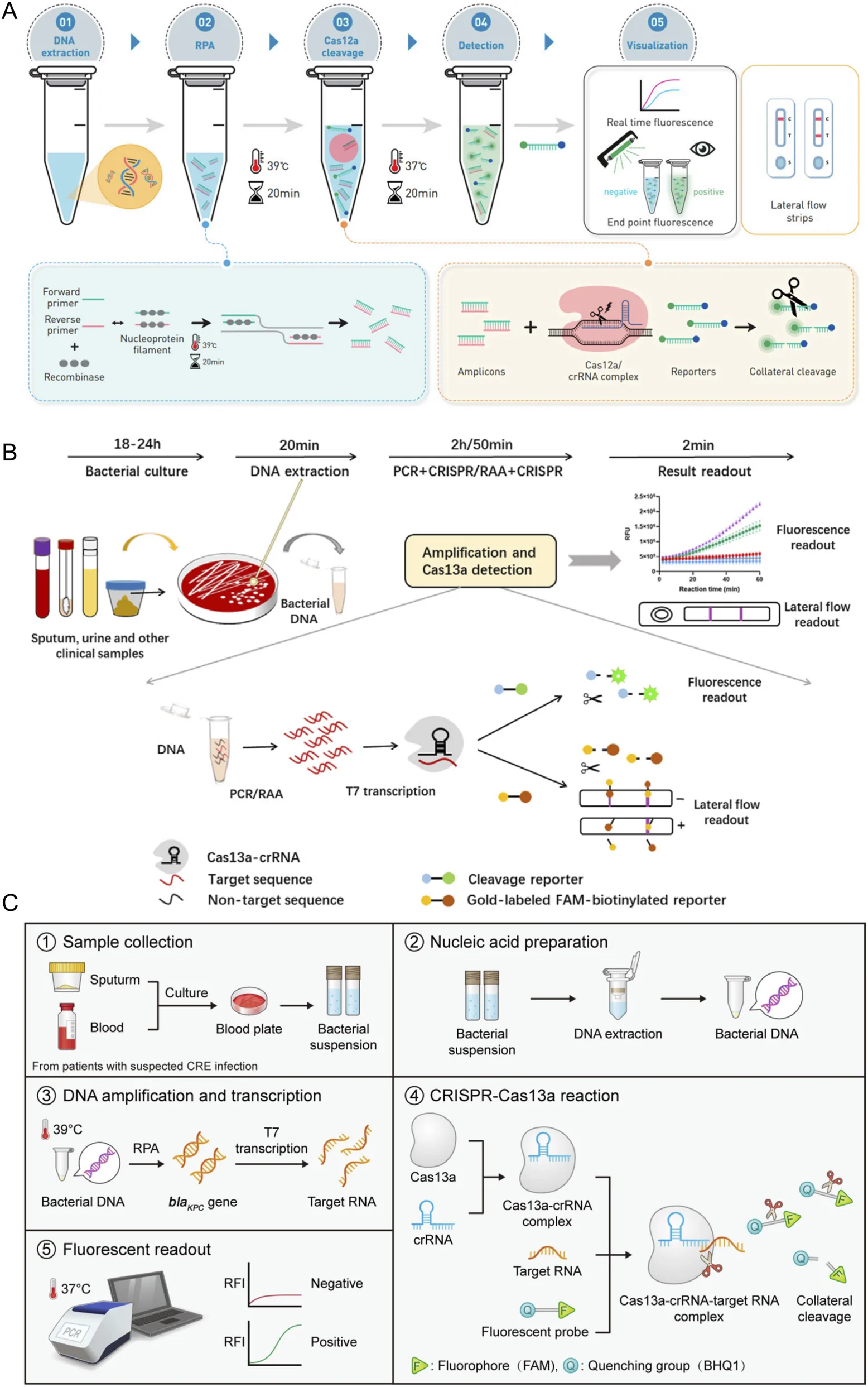
Representative CRISPR/Cas workflows for carbapenemase gene detection. **(A)** The recombinase polymerase amplification-coupled CRISPR/Cas12a system (RCCS) amplifies *blaKPC* and *blaNDM* after DNA extraction and then uses target-specific Cas12a recognition. Adapted from Yang et al. (Yang et al., 2024). Copyright © 2024 by the authors; distributed under the terms of the Creative Commons Attribution 4.0 International License (CC BY 4.0). **(B)** PCR- or RAA-assisted Cas13a testing combines T7 transcription and Cas13a collateral cleavage to detect *Klebsiella pneumoniae* together with *blaKPC* and *blaNDM*. Reproduced from Cao et al. (Cao et al., 2024). Copyright © 2023 Taiwan Society of Microbiology; published by Elsevier Taiwan LLC under the Creative Commons Attribution-NonCommercial-NoDerivatives 4.0 International License (CC BY-NC-ND 4.0). **(C)** The RPA–T7–Cas13a assay for *blaKPC* uses RPA amplification, T7 transcription of the amplified target, crRNA-guided Cas13a recognition, and collateral cleavage of a FAM/BHQ1 RNA reporter for real-time fluorescence detection. Adapted from Liang et al. (Liang et al., 2023). Copyright © The Author(s) 2023; distributed under the terms of the Creative Commons Attribution 4.0 International License (CC BY 4.0).

Carbapenemase-targeted CRISPR/Cas studies can be viewed as a progression from single-gene analytical confirmation toward broader target coverage and more integrated workflows. Representative carbapenemase-detection architectures include an RPA–Cas12a workflow (Figure 4A), a PCR/RAA–T7–Cas13a workflow (Figure 4B), and an RPA–T7–Cas13a workflow (Figure 4C). Early Cas12a and Cas13a platforms established the feasibility of detecting *blaKPC*, *blaNDM*, *blaOXA-48-like*, and related determinants using fluorescence or lateral-flow readouts, but most early evaluations were isolate-based or analytically focused (Curti et al., 2020; Xu et al., 2022; Li et al., 2024; Gao et al., 2025; Shin et al., 2024; Liang et al., 2023; Patil et al., 2025). These studies support sequence-specific carbapenemase detection, not complete CRKP phenotyping.

Multi-target coverage is more closely aligned with clinical screening needs but requires careful interpretation of the underlying validation set. Xu et al. (Xu et al., 2024) evaluated an RPA–Cas12a workflow targeting KPC, NDM, OXA, IMP, and VIM in a reported 75-item clinical evaluation and reported complete concordance with conventional PCR. However, the source article describes the evaluated material inconsistently as clinical strains and sputum specimens and contains minor inconsistencies in subgroup totals. We therefore do not reproduce the uncertain individual subgroup counts and interpret the study primarily as evidence of multi-target analytical and clinical feasibility rather than as a definitive diagnostic-performance estimate.

One-pot and near-patient workflows represent a further engineering step. Hyeon et al. (Hyeon et al., 2026) used target-specific suboptimal PAMs to delay Cas12a activation in a one-pot RPA–Cas12a system. Under the reported 30-min analytical reaction, the LODs were 10^−17^ M for *blaKPC* and 10^−16^ M for *blaNDM*. Clinical evaluation included 44 rectal swab specimens subjected to a 10-min boiling pretreatment followed by the 30-min one-pot assay; the authors reported 100% sensitivity and 100% specificity relative to multiplex qPCR, corresponding to a reported sample-to-answer time of approximately 40 min. These estimates should nevertheless be interpreted in the context of the limited single-matrix cohort and require external validation.

Detection of a carbapenemase gene does not by itself establish a complete CRKP phenotype and should be reported as a carbapenemase-associated resistance signal unless organism linkage and phenotypic resistance are independently demonstrated (Tamma and Simner, 2018). Carbapenemase-producing and non-carbapenemase-producing carbapenem-resistant Enterobacterales (CRE) bacteremia can differ in clinical outcomes, reinforcing the value of distinguishing mechanism-level detection from broader phenotypic classification (Tamma et al., 2017). Presence/absence-based gene panels are best suited to defined, sequence-identifiable mechanisms such as major carbapenemases and selected ESBL or mobile resistance genes. By contrast, carbapenem resistance may also arise through porin loss combined with ESBL/AmpC activity, efflux or regulatory changes, insertional effects, copy-number variation, and other multilocus or expression-dependent mechanisms that are not captured reliably by a fixed short-target panel (Li Y. et al., 2023; Bialek-Davenet et al., 2017). Molecular results should therefore be interpreted with specimen context, phenotypic AST, and local epidemiology, with sequencing considered when genotype–phenotype discordance or mechanism resolution is clinically important (Tamma and Simner, 2018; Li Y. et al., 2023; Bialek-Davenet et al., 2017).

### 4.2 Detection of ESBL genes, other β-lactamase genes, and additional ARGs

In settings where rapid carbapenemase detection is the immediate priority, ESBL genotyping may have a secondary role; its value nevertheless depends on specimen type, local epidemiology, and antimicrobial stewardship objectives. Wang et al. (Wang et al., 2023) developed a PCR–LbCas12a platform that detected a Kp-associated target and a *blaSHV*-family target and evaluated it using sputum specimens and clinical Kp isolates. Vargas-Reyes et al. (Vargas-Reyes et al., 2026) developed the C12a toolbox for *blaCTX-M-15*, *floR*, and *intI1* detection. In the reported isolate sets, *blaCTX-M-15* was detected in seven ESBL-producing isolates and was absent from eight β-lactam-susceptible isolates; *floR* calls were concordant with the corresponding resistance characterization in 11 positive and six negative isolates; and *intI1* was detected in 17 of 18 ARG-positive *E. coli* isolates. These small proof-of-concept cohorts support target-level feasibility but do not establish broad diagnostic performance. Tyumentseva et al. (Tyumentseva et al., 2025) reported detection of a model-template input of 1.25 copies per reaction for *blaOXA-1*, but this analytical result was obtained after a 65-min conventional PCR preamplification step followed by approximately 5 min of Cas12a detection, giving a total assay time of approximately 70 min. The optimized workflow was subsequently evaluated using 50 WGS-confirmed *blaOXA-1*-positive and 11 *blaOXA-1*-negative genomic DNA samples. Accordingly, this result is more accurately described as amplification-assisted near-single-copy analytical detection rather than direct specimen-level single-copy sensitivity. Together, these studies illustrate antimicrobial resistance gene (ARG) detection across different validation contexts, but their clinical interpretation remains target and specimen dependent.

These studies suggest that a Kp ESBL panel may combine conserved family-level screening with confirmation of clinically important subtypes. Cas12a can serve not only as a fluorescence readout after amplification but also as a secondary sequence-confirmation layer for PCR products, reducing misinterpretation caused by nonspecific amplification (Li et al., 2018). The boundary between ARG detection and phenotypic resistance is particularly important because alleles within the *blaSHV* and *blaTEM* families can have different hydrolysis spectra. A single positive result should therefore not be interpreted as a specific susceptibility phenotype. Genotypic CRISPR/Cas panels are better suited to providing an early indication of resistance risk than to complete susceptibility prediction. Resistance caused by regulatory mutations, porin alterations, efflux-pump upregulation, or insertional inactivation still requires AST, targeted sequencing, or WGS (Hujer et al., 2020; Ellington et al., 2017; Yap et al., 2022).

The broader Kp resistome and mobilome further limit the completeness of fixed target panels. Genomic diversity includes allele variation, SNPs and indels, insertion sequences, integrons, recombination, plasmid exchange, and other horizontally transferred elements that can alter both resistance-mechanism prevalence and CRISPR target conservation. A recent *in silico* analysis of β-lactamase-producing *K. pneumoniae* illustrates the breadth of resistome, virulome, and mobilome diversity and supports periodic genome-informed reassessment of panel coverage (Biswas and Anbarasu, 2026). Redundant crRNAs directed at independent conserved regions may reduce single-site variant-associated dropout, but each component and the combined panel still require inclusivity, competition, and discordance testing; multiplex design cannot be assumed to eliminate genomic false negatives.

### 4.3 Detection of colistin- and tigecycline-resistance determinants

**FIGURE 5:**
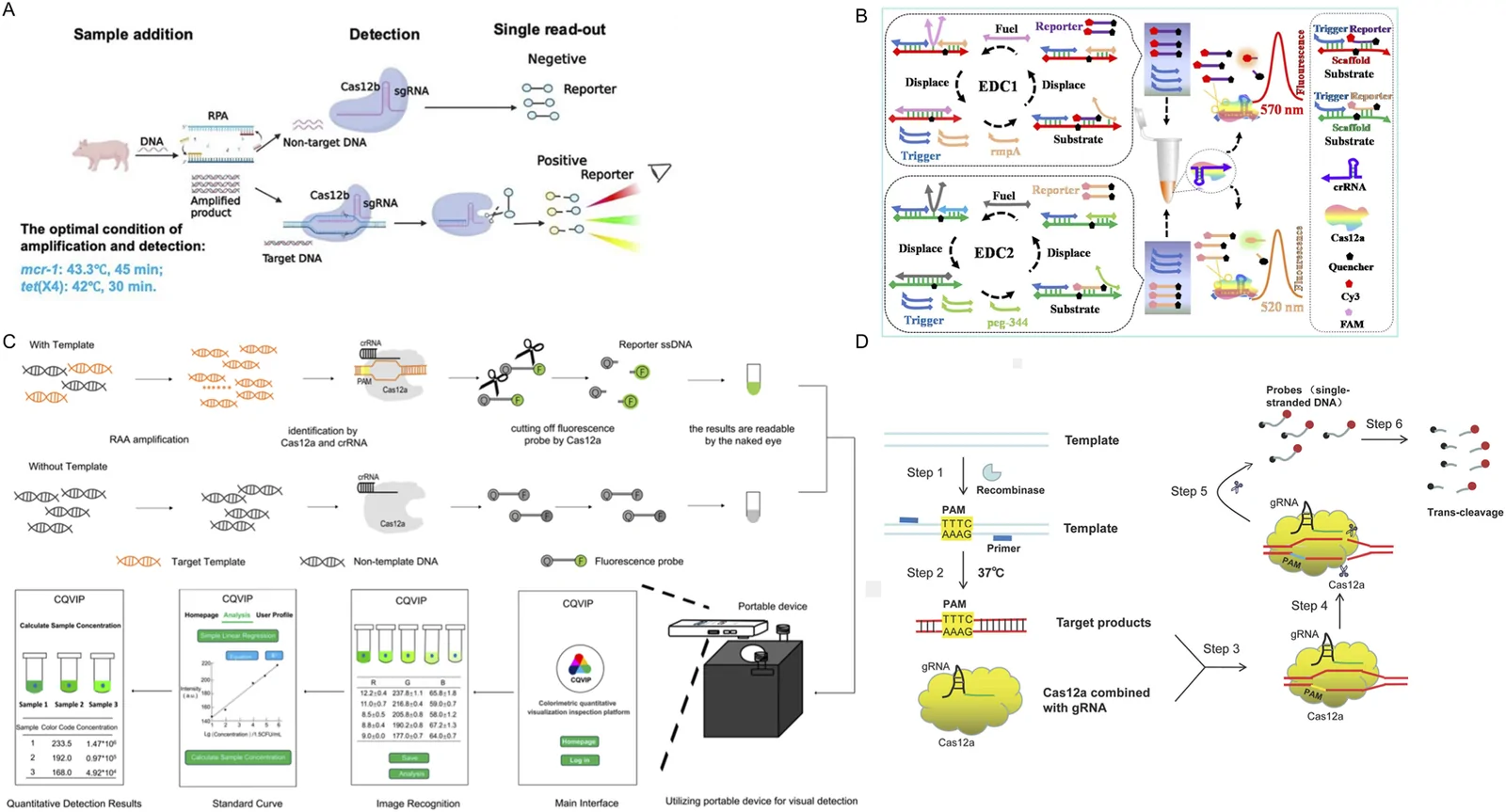
Representative CRISPR/Cas strategies for detecting resistance to last-line antimicrobials and hypervirulence-associated markers. **(A)** A one-tube RPA–CRISPR/Cas12b workflow combines sample DNA with target-specific RPA and Cas12b reagents for rapid detection of *mcr-1* or *tet(X4)*. Adapted from Wang et al. (Wang et al., 2024). Copyright © The Author(s) 2024; distributed under the terms of the Creative Commons Attribution 4.0 International License (CC BY 4.0). **(B)** Two parallel entropy-driven circuits separately recognize *rmpA* and *peg-344* and release a shared trigger together with target-specific Cy3-or FAM-labeled reporters. Reproduced with permission from Long et al. (Long et al., 2025). Permission conveyed through Copyright Clearance Center, Inc. **(C)** The Cas12a smartphone-assisted colorimetric quantitative platform couples RAA, crRNA-guided Cas12a recognition, and reporter cleavage with a portable imaging device and the CQVIP smartphone application. Reproduced from Huang et al. (Huang et al., 2026). Copyright © 2026 The Authors; published by the American Chemical Society under the Creative Commons Attribution-NonCommercial-NoDerivatives 4.0 International License (CC BY-NC-ND 4.0). **(D)** The RPA–CRISPR/Cas12a assay for *mcr-1* first amplifies a target region containing a compatible PAM and then uses gRNA-guided Cas12a recognition to activate collateral cleavage of fluorescent ssDNA probes, providing a rapid fluorescence readout. Adapted from Gong et al. (Gong et al., 2022). Copyright © 2022 Gong et al.; distributed under the terms of the Creative Commons Attribution 4.0 International License (CC BY 4.0).

Studies of *mcr-1* and *tet(X4)* provide methodological evidence for surveillance of resistance to last-line antimicrobials. Gong et al. (Gong et al., 2022) reported an analytical LOD of 420 fg DNA for the *mcr-1* RPA–Cas12a assay. In spiked matrices, the reported organism-level detection thresholds were approximately 6.2 × 10^3^ CFU/mL in fecal samples, 2.6 × 10^3^ CFU/mL in blood, and 1.6 × 10^3^ CFU/mL in urine, corresponding to approximately 6.2, 2.6, and 1.6 CFU per reaction, respectively, under the study-specific input conditions (Figure 5D). Wang et al. (Wang et al., 2024) developed a one-tube RPA–Cas12b assay for *mcr-1* and *tet(X4)*, achieving LODs of 6.25 and nine copies, respectively, with total assay times of approximately 55 and 40 min. The assay was evaluated using pork and market environmental samples (Figure 5A).

For Kp, detection of an *mcr* gene can provide an early warning of mobile colistin resistance (Liu et al., 2016; Sun et al., 2018). However, colistin resistance in Kp is also commonly caused by chromosomal mutations or regulatory alterations that cannot be inferred from the presence or absence of a single conserved target. These mechanisms involve multilocus variation and insertion events (Yap et al., 2022; Yang et al., 2020) and are better addressed through mutation-specific crRNA combinations, sequencing, or CRISPR-based single-nucleotide variant (SNV) recognition (Li et al., 2018). *tet(X)* genes are more appropriate as markers of mobile tigecycline resistance in Enterobacterales and One Health surveillance than as universal core targets in clinical Kp panels (He et al., 2019). Reports concerning last-line resistance should therefore describe gene-associated risk and recommend interpretation in conjunction with AST and the locally prevalent resistance-mechanism spectrum.

### 4.4 Detection of hypervirulence-associated markers

Detection of hypervirulence-associated markers is an emerging focus of CRISPR/Cas research on Kp. Li et al. (Li C. et al., 2023) combined violet phosphorene nanosheets with RPA–Cas12a to detect hypervirulence-associated genes and *tet(A)* and used smartphone-based fluorescence imaging to reduce background signals. Bhattacharjee et al. (Bhattacharjee et al., 2024) developed a paper-based RPA–Cas13a assay targeting a Kp-associated marker and *rmpA*, providing early evidence for minimal-instrumentation screening for hypervirulence-associated risk. Huang et al. (Huang et al., 2026) constructed a smartphone-assisted colorimetric platform for semiquantitative detection of multiple hypervirulence-associated genes by combining RGB analysis with a portable device (Figure 5C).

Long et al. (Long et al., 2025) used entropy-driven circuits to regulate a single Cas12a system and distinguish *rmpA* from *peg-344*, addressing the multiplex-encoding challenge created by shared trans-cleavage (Figure 5B). Collectively, these studies have advanced hypervirulence-associated risk screening through lower-background detection, paper-based operation, smartphone colorimetry, and nucleic acid circuit encoding. However, the markers are not interchangeable. Aerobactin loci, including *iucABCD*/*iutA*, and the virulence-plasmid-associated biomarker *peg-344* may be considered candidate priority markers for conceptual panel design because they have been repeatedly associated with hypervirulent lineages in the cited cohorts (Russo et al., 2018; Bulger et al., 2017). Detection of *rmpA*/*rmpA2* can provide Supplementary Material on capsule regulation or a hypermucoviscosity-associated genetic profile (Russo et al., 2018; Cheng et al., 2010; Lin et al., 2019), but short-target assays do not establish full-locus integrity, absence of truncation, or expression. The *iro* locus, K1/K2 capsular types, and clonal backgrounds such as ST23 (Lam et al., 2018) or ST11 (Gu et al., 2018) are better reported as contextual modifiers than as stand-alone criteria for hvKp (Russo and Marr, 2019). This proposed marker prioritization is conceptual and requires prospective validation.

A hypervirulence-associated genotype should be distinguished from an experimentally demonstrated hypervirulence phenotype. Short-target detection does not establish locus integrity, expression, siderophore production, capsule-regulatory function, serum resistance, or virulence in an appropriate experimental model. Prospective validation of molecular hvKp classification should therefore relate multi-marker profiles to expression or functional phenotypes, microbiological findings, compatible clinical syndromes, and, when appropriate, genomic and experimental virulence evidence. These requirements are particularly important for isolates with acquired resistance, in which the relationship between canonical virulence markers and measured virulence may be heterogeneous (Russo et al., 2024; Kochan et al., 2023).

CRISPR/Cas testing for hvKp is therefore better positioned as a screening approach for hypervirulence-associated risk than as a stand-alone diagnostic test for confirmed hvKp. Future clinical reporting platforms should use multi-marker combinations, distinguish priority virulence-associated targets from contextual modifiers, and state the interpretive weight and misclassification risk of each category. Reports should avoid the statement “hvKp detected” on the basis of a single marker and should instead use bounded wording such as “hypervirulence-associated marker(s) detected; classification requires correlation with the clinical syndrome, culture findings, and genomic evidence.”

### 4.5 PAM engineering, PAM independence, and target adaptation

Target design for CRISPR/Cas detection is often constrained by PAM requirements, particularly when multiple resistance or virulence genes must be included. Wu et al. (Wu et al., 2026) developed PEAR, which embeds an engineered PAM during RPA through an Nfo-cleavable AP-site probe and therefore reduces dependence on a suitable native PAM adjacent to the target sequence. Sanger sequencing and controlled PAM-present/PAM-absent experiments confirmed incorporation and functional activity of the engineered PAM. Using plasmid-derived templates, the assay achieved an analytical LOD of one copy/μL for both *khe* and *blaKPC-2*, with 100% analytical specificity in the tested panel. In 30 clinical sputum specimens, the main Results and Figure 7 report diagnostic accuracies of 93.3% for *khe* and 86.7% for *blaKPC-2* relative to qPCR. Notably, the source abstract reports 87.7% for *blaKPC-2*, whereas the main Results, contingency data, and Conclusion support 86.7%; we therefore use the latter value. These estimates remain preliminary because of the small single-center cohort and require confirmation in larger external studies.

The importance of PAM engineering extends beyond improving a single target and includes expanding the available design space for multi-target panels. Not all markers at different functional levels contain ideal natural PAMs (Wu et al., 2026; Collias and Beisel, 2021), and strict dependence on PAM position can constrain assay design and produce target-specific performance differences. The engineered-PAM strategies used in PEAR and CRISPR-KP show that PAM availability can be engineered through amplification or probe design rather than being treated as a fixed constraint imposed by the native target sequence (Wu et al., 2026). However, these approaches increase the complexity of probe, enzyme, and reaction optimization. Clinical translation still requires assessment of lot-to-lot stability and resistance to interference in complex specimens (Burd, 2010).

Across Section 4, the translational value of CRISPR/Cas lies less in detecting any single resistance or virulence marker than in combining organism context with clinically selected target categories and reporting each result within its interpretive boundary. Combining organism-context and resistance-associated targets may support earlier AST or stewardship review; hypervirulence-associated targets may justify syndrome correlation and isolate/genomic evaluation; and co-detection of resistance and candidate priority hypervirulence markers may trigger a confirmatory convergence workflow without proving same-strain carriage. This combination-to-report logic is developed explicitly in Section 5.

## 5 Multiplex detection, target panels, and resistance–hypervirulence risk reporting

### 5.1 Multiplex CRISPR/Cas is not simply the addition of more targets

Multiplex detection is important for moving Kp CRISPR/Cas platforms from analytical feasibility toward clinical evaluation, but the challenge extends beyond adding multiple crRNAs to one workflow. Primer and crRNA concentrations, target abundance, target copy number, amplification efficiency, shared reagent consumption, collateral cleavage, reporter competition, cross-channel interference, and concentration imbalance can create target-dependent kinetics. High-abundance or efficiently amplified targets may dominate the reaction and increase the risk of low-copy target dropout, whereas indiscriminate trans-cleavage may complicate target attribution when reporters are not sufficiently separated. Each target should therefore be evaluated alone and in clinically plausible mixtures across a range of concentration ratios, with prespecified criteria for competition, dropout, and indeterminate results. Spatial separation, digital partitioning, multichannel reporting, or nucleic acid circuit encoding may improve target resolution, but these approaches add cost and workflow complexity.

**FIGURE 6:**
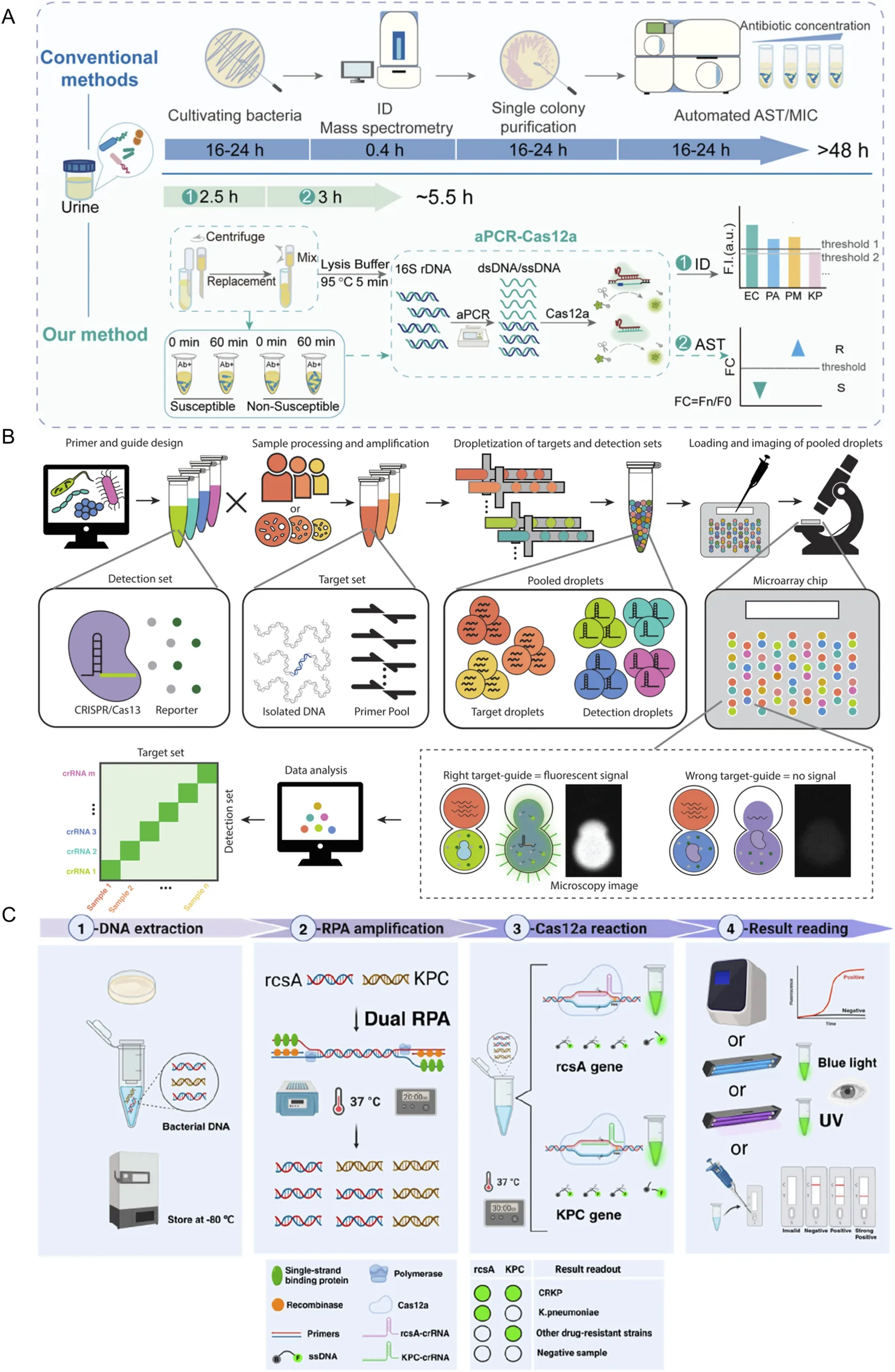
Representative CRISPR/Cas platforms connecting broad multiplex detection, resistance-risk screening, and phenotypic antimicrobial susceptibility testing. **(A)** The asymmetric PCR–Cas12a platform performs direct bacterial identification and rapid phenotypic AST from urine. Adapted with permission from Zheng et al. (Zheng et al., 2025). Copyright 2025 American Chemical Society. **(B)** The bCARMEN workflow combines pooled target amplification with Cas13 detection sets, droplet microarray pairing, fluorescence microscopy, and computational analysis to support highly multiplexed detection of bacterial species and antimicrobial resistance genes. Adapted from Thakku et al. (Thakku et al., 2022). Copyright © 2022 The Author(s); published by Oxford University Press on behalf of the National Academy of Sciences under the Creative Commons Attribution 4.0 International License (CC BY 4.0). **(C)** A dual RPA–CRISPR/Cas12a workflow co-amplifies *rcsA* and *blaKPC* from extracted bacterial DNA, followed by separate target-specific Cas12a reactions. Adapted from Tan et al. (Tan et al., 2024a). Copyright © 2024 Tan et al.; distributed under the terms of the Creative Commons Attribution License (CC BY).

Existing multiplex CRISPR/Cas strategies can be considered at two levels. Beyond genotypic detection, CRISPR/Cas platforms can also integrate direct bacterial identification with rapid phenotypic AST, as illustrated in Figure 6A. At the broad-panel level, Cas13-based droplet-array platforms such as bCARMEN show that CRISPR diagnostics can scale from single-pathogen detection to panels covering multiple bacterial species and ARGs (Thakku et al., 2022) (Figure 6B). These platforms demonstrate multiplex scalability, but their liquid-handling, imaging, and computational requirements also show why high target capacity does not automatically translate into near-patient use. At the Kp-oriented level, more focused workflows have incorporated Kp-associated targets, carbapenemase genes, or hypervirulence-associated markers (Cao et al., 2024; Xiang et al., 2024; Xie et al., 2025; Xu et al., 2024; Huang et al., 2026; Long et al., 2025). For example, dual RPA–Cas12a detection of Kp *rcsA* and *blaKPC* links organism-context detection with resistance gene screening in one workflow (Tan et al., 2024a) (Figure 6C). Because the amplified products still require target-specific Cas12a readouts, this approach represents a step toward dual-target reporting rather than a fully integrated single-tube multiplex interpretation system.

Taken together, broad-panel and Kp-oriented studies show that multiplexing can be achieved through reaction compartmentalization, spatial separation, reporter encoding, microfluidic partitioning, or combinations of Cas effectors. Its clinical value depends less on the absolute number of targets than on whether the target combination addresses a predefined diagnostic question. A multipathogen panel without resistance targets may support etiological assessment but cannot define susceptibility. A resistance gene panel without a validated Kp/KpSC-associated target cannot provide Kp-related organism context. Likewise, a virulence-marker panel without resistance information cannot flag possible resistance–hypervirulence convergence.

For Kp, multiplex detection should prioritize interpretability rather than target count. A Kp-oriented panel may include at least one validated Kp-associated or KpSC-associated target, one or more locally prevalent carbapenemase targets or other resistance-associated targets, and candidate priority hypervirulence-associated markers. The value of multiplexing lies in generating bounded outputs—organism-context detection, gene-associated resistance risk, hypervirulence-associated risk, or a suspected resistance–hypervirulence convergence risk signal.

### 5.2 Principles for scenario-driven target panel design

A stratified target framework should not be interpreted as a universal panel recommendation or applied mechanically across patients and specimens. Candidate targets should be selected according to global and regional prevalence, local epidemiology, clinical impact, allele diversity, expected diagnostic coverage, specimen type, therapeutic or infection control relevance, and the action that a positive result is intended to trigger. Major carbapenemases may merit core consideration where they are prevalent because they represent clinically important sequence-identifiable resistance determinants, whereas ESBL, last-line resistance, clonal, mobile-element, or hypervirulence-associated targets should be added only when their local prevalence and intended use justify inclusion. Target selection should be revisited as circulating lineages and resistance mechanisms change, and the analytical inclusivity of each target should be established against contemporary genome collections before clinical validation. A scenario-specific matrix of candidate target content for prospective validation is provided in Supplementary Table S3.

### 5.3 Conceptual four-layer target-to-report framework

For prospective evaluation, a conceptual Kp CRISPR/Cas target-to-report framework can be organized into four layers. The first layer establishes Kp-associated or KpSC-associated organism context using analytically validated targets, with explicit caution when closely related KpSC members have not been excluded (McAndrew et al., 2025; Long et al., 2017). The second layer detects locally relevant resistance-associated targets, including carbapenemase genes such as *blaKPC*, *blaNDM*, *blaOXA-48-like*, *blaIMP*, and *blaVIM*, together with selected ESBL genes or last-line resistance markers when epidemiologically justified (Xu et al., 2024; Liu et al., 2016; Simner et al., 2024). The third layer prioritizes candidate hypervirulence-associated markers such as the aerobactin loci (*iucABCD*/*iutA*) and *peg-344*, with *rmpA*/*rmpA2* retained as supporting targets; full-locus integrity requires multi-region or sequence-level confirmation. The *iro* locus, K1/K2 capsular types, and clonal backgrounds such as ST23 or ST11 should be reported separately as contextual modifiers (Gu et al., 2018; Lam et al., 2018). The fourth layer integrates the co-detection pattern with specimen type and testing context to flag possible resistance–hypervirulence convergence without implying same-strain carriage or same-plasmid colocalization (David et al., 2020; Arcari and Carattoli, 2023).

Framework layers define what is measured; reporting stages define how combinations are interpreted. Routine screening may require only Kp- or KpSC-associated organism-context detection and core carbapenemase targets, whereas ICU pneumonia, invasive community-onset infection, or suspected liver abscess may justify prospective evaluation of additional hypervirulence-associated or last-line resistance markers (Lan et al., 2021; He et al., 2019; Arcari and Carattoli, 2023). Outbreak investigation may additionally justify the inclusion of genomic-background markers whose significance can subsequently be resolved through clonal and plasmid analysis (David et al., 2020; Lam et al., 2018). The objective is not to maximize target count but to ensure that each target category corresponds to a predefined interpretation and confirmatory pathway (Ellington et al., 2017). The conceptual four-layer target-to-report framework is summarized in Table 2.

**TABLE 2:** Conceptual four-layer target-to-report framework for prospective evaluation of Kp/KpSC risk reporting.

Layer	Target groups	Candidate interpretive output	Interpretive boundary and potential confirmation
Layer 1: organism context	Assay-specific Kp sensu stricto or KpSC-associated targets, such as validated *rcsA*, *khe*, or *rpoB* regions or other discriminatory assays	“Kp-associated nucleic acid detected” or “KpSC-associated target detected,” according to demonstrated analytical specificity	Do not claim *K. pneumoniae* sensu stricto when closely related KpSC members have not been excluded; confirm with culture/MALDI-TOF or genomic methods when needed
Layer 2: resistance-associated risk	Locally prevalent carbapenemases (*blaKPC*, *blaNDM*, *blaOXA-48-like*, *blaIMP*, *blaVIM*); selected ESBLs and last-line resistance markers when epidemiologically justified	Carbapenemase-, ESBL-, mobile-colistin-, or *tet(X)*-associated resistance risk; organism attribution requires appropriate linkage evidence	Detection of a resistance gene does not constitute a complete antimicrobial susceptibility phenotype. In mixed specimens, co-detection with a Kp/KpSC-associated target does not establish that the resistance marker is carried by Kp. Perform AST and consider sequencing for mechanisms outside the panel
Layer 3: hypervirulence-associated risk	Consider aerobactin loci (*iucABCD*/*iutA*) and *peg-344* as candidate priority markers; use *rmpA*/*rmpA2* target detection as Supplementary Material, with locus integrity requiring sequence-level confirmation. Report *iro*, K1/K2, ST23/ST11, and other backgrounds separately as contextual modifiers	“Hypervirulence-associated marker(s) detected, indicating a hypervirulence-associated risk signal”	A single marker, K1/K2, ST23, *magA*, or a string test cannot independently define hvKp; integrate syndrome, phenotype, and genomic evidence
Layer 4: integrated convergence and background	Co-detection pattern plus specimen source, organism purity, and clonal or mobile-element information when independently available	“Suspected resistance–hypervirulence convergence risk signal”	Specimen-level co-detection does not prove same-strain carriage or same-plasmid colocalization. Consider isolate recovery, AST, isolate preservation, and WGS/plasmid analysis according to validated local workflows

The four layers are not intended to imply equal marker weighting or fixed numerical decision thresholds. Each target has a distinct biological meaning and analytical evidence base. Organism-context targets establish the scope of attribution; resistance markers contribute only target-specific gene-associated risk; candidate priority hypervirulence markers and contextual modifiers should not be treated as interchangeable; and convergence-related reporting depends on the strength of organism linkage. Before clinical use, each proposed rule should be prospectively locked and validated in an intended-use cohort using prespecified target combinations, invalid/discordant rules, reference methods, and clinical or workflow endpoints. Calibration should estimate target-specific likelihood ratios or other measures of incremental information and determine whether adding a marker improves actionable classification rather than merely increasing panel size.

### 5.4 Risk-oriented reporting framework

This reporting framework is conceptual rather than a validated clinical score or independent decision rule. Its purpose is not to infer disease severity from CRISPR/Cas results, but to translate multi-target molecular findings into bounded language and identify confirmation steps that may be considered within locally validated workflows. The reporting stages sequentially integrate Kp-associated or KpSC-associated organism context, resistance- and hypervirulence-associated targets, and genetic background information when available. Each stage must be interpreted in the context of the specimen source, the likelihood of infection versus colonization, phenotypic AST, genomic findings, and local epidemiology. Prospective cohorts should calibrate the framework against phenotypic, genomic, workflow, and clinical-outcome data.

For terminology, three evidence levels should be distinguished. “Specimen-level co-detection” denotes concurrent detection of relevant targets without organism linkage. “Probable same-organism association” may be used only when independent microbiological evidence strongly supports a dominant or apparently monomicrobial Kp context but same-strain genomic confirmation is absent. “Confirmed same-strain resistance–hypervirulence convergence” requires a recovered isolate or single-colony lineage with appropriate evidence of organism identity, resistance, hypervirulence-associated markers, and genomic linkage; plasmid-level colocalization requires plasmid-resolved evidence. Primary or mixed specimens should therefore remain classified at the specimen-level co-detection or suspected-risk level unless stronger linkage evidence is available.

The present framework is deliberately categorical because the available studies do not provide sufficiently standardized likelihood ratios for a validated probabilistic score. In principle, however, molecular results could be integrated with pretest probability and local prevalence using Bayesian updating once target-specific likelihood ratios have been established prospectively. The same sensitivity and specificity can yield very different predictive values across settings. For illustration, a hypothetical assay with 95% sensitivity and 95% specificity would have a positive predictive value of approximately 16% at 1% prevalence but approximately 83% at 20% prevalence, whereas the negative predictive values would be approximately 99.95% and 98.7%, respectively. These values are illustrative rather than performance estimates for a specific Kp assay. Future validation should therefore report prevalence, positive predictive value (PPV), negative predictive value (NPV), and, where appropriate, likelihood ratios rather than relying on sensitivity and specificity alone.

#### 5.4.1 Organism context

After assay validity has been established and predefined thresholds have been applied, detection of a Kp-associated or KpSC-associated target alone may be reported as “Kp-associated or KpSC-associated nucleic acid detected; resistance and hypervirulence markers included in this panel were not detected.”

#### 5.4.2 Resistance-associated risk

In a cultured isolate or a specimen with independently established organism linkage, co-detection of an organism-context target and *blaKPC*, *blaNDM*, or another validated carbapenemase marker may be reported as indicating carbapenemase-associated resistance risk and may prompt phenotypic AST or infection control review. In a mixed or non-isolate specimen, the report should state that the targets were co-detected at the specimen level and that Kp carriage of the resistance marker has not been established.

#### 5.4.3 Hypervirulence-associated risk

Detection of candidate priority markers such as *iucABCD*/*iutA* or *peg-344*, with or without supporting *rmpA*/*rmpA2* detection, may be reported as a hypervirulence-associated risk signal; classification requires correlation with the clinical syndrome, culture findings, and genomic evidence.

#### 5.4.4 Suspected convergence

**FIGURE 7:**
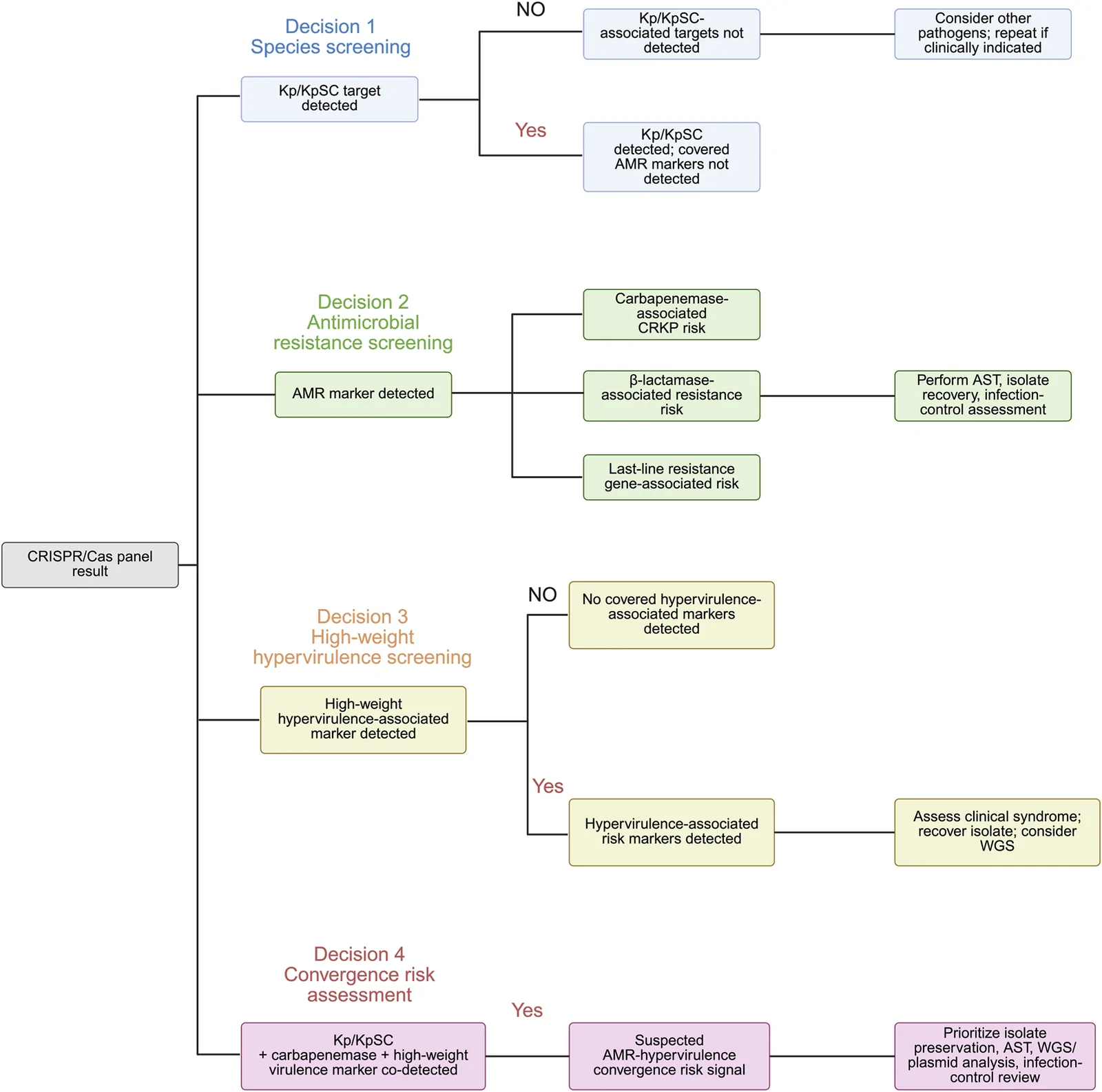
Conceptual four-stage framework for risk-oriented interpretation of CRISPR/Cas panel results in *Klebsiella pneumoniae*/KpSC testing. (Stage 1) Apply predefined validity criteria and assess a validated Kp-associated or KpSC-associated target. (Stage 2) Categorize detected resistance markers as gene-associated risk and link them to phenotypic AST or other confirmation as appropriate. (Stage 3) Report candidate priority hypervirulence-associated markers as a bounded risk signal that requires clinical, microbiological, and genomic correlation. (Stage 4) Report co-detection of an organism-context target, a carbapenemase marker, and a candidate priority hypervirulence-associated marker as a suspected resistance–hypervirulence convergence risk signal; specimen-level co-detection does not establish same-strain or same-plasmid carriage. All follow-up actions depend on the specimen context and a locally validated workflow.

Co-detection of an organism-context target, a carbapenemase marker, and one or more candidate priority hypervirulence-associated markers may be reported as a suspected resistance–hypervirulence convergence risk signal. Isolate recovery, AST, isolate preservation, and WGS or plasmid-level analysis may be considered according to the clinical context and local workflow. The four reporting stages are summarized in Figure 7.

This distinction is particularly important in non-isolate samples, mixed infections, and colonization-screening specimens. Simultaneous positivity for an organism-context target, resistance markers, and hypervirulence-associated markers represents specimen-level co-detection and does not prove that the markers are present in the same Kp strain or on the same genetic vehicle. Unless testing is performed on a pure cultured isolate or same-strain carriage is demonstrated by single-colony isolation and genomic evidence, the report should use only the term “suspected resistance–hypervirulence convergence risk signal.” An apparently monomicrobial positive blood culture bottle increases, but does not establish, the likelihood of same-organism carriage. Table 3 summarizes the detection-pattern-to-report logic, and Supplementary Notes one and two provide operational definitions and example wording.

**TABLE 3:** Conceptual detection-pattern-to-report framework for Kp/KpSC CRISPR/Cas panels.

Molecular pattern	Suggested report wording	Potential follow-up within a locally validated workflow	Interpretive caveat
Kp-associated or KpSC-associated target detected; covered resistance and hypervirulence markers not detected	Kp/KpSC-associated nucleic acid detected; resistance and hypervirulence markers included in this panel were not detected	Culture/identification and AST as clinically indicated	A negative panel result indicates only that the tested targets were not detected within the validated analytical limits. It does not exclude mechanisms outside the panel, known variant gaps, low organism burden, or colonization/infection uncertainty in non-sterile specimens
Kp-associated or KpSC-associated target plus carbapenemase marker(s)	A Kp/KpSC-associated target and carbapenemase marker(s) were co-detected. In an organism-linked isolate, this pattern indicates carbapenemase-associated resistance risk; in a mixed specimen, Kp carriage is not established	Consider phenotypic AST, infection prevention assessment, and isolate preservation; consider WGS during outbreaks or unusual phenotypes according to validated local workflows	Do not equate gene detection with a complete CRKP phenotype. In mixed or non-isolate specimens, specimen-level co-detection does not establish organism linkage; non-carbapenemase mechanisms and genotype–phenotype discordance also remain possible
Kp-associated or KpSC-associated target plus ESBL or other resistance marker(s), without a carbapenemase target	The specified resistance-associated marker(s) were detected; interpretation requires allele-level, organism-linkage, and phenotypic context	AST and antimicrobial stewardship review; interpret against local allele prevalence and panel scope	Family-level genes such as *blaSHV*/*blaTEM* may include alleles with different hydrolysis spectra
Kp-associated or KpSC-associated target plus candidate priority hypervirulence-associated marker(s)	Hypervirulence-associated marker(s) were detected; correlation with clinical and microbiological findings is required	Assess for a compatible clinical syndrome, recover an isolate, and consider virulence phenotyping/WGS.	Do not report “hvKp detected” from a single marker
Kp-associated or KpSC-associated target plus carbapenemase marker(s) and candidate priority hypervirulence-associated marker(s)	Suspected resistance–hypervirulence convergence risk signal warranting confirmatory evaluation according to validated local workflows	Consider isolate recovery, AST, infection control risk assessment, isolate preservation, and WGS/plasmid analysis according to validated local workflows	In mixed or non-isolate specimens, this is specimen-level co-detection and does not establish carriage in the same strain or colocalization on the same genetic vehicle
Resistance or virulence marker detected without a Kp-associated or KpSC-associated organism-context target	Target detected without confirmed Kp/KpSC context	Repeat or orthogonal testing, culture, and assessment for another organism, contamination, or extracellular/free nucleic acid	Do not attribute the marker to Kp without organism linkage
Internal/process control failure or result outside predefined validity rules	Invalid result; no clinical interpretation	Repeat testing from the same extract when appropriate, or recollect and reprocess the specimen	Failure of a required specimen-adequacy, extraction/process, amplification, Cas/reporter, or inhibition control should invalidate the result rather than support a negative interpretation. Ambiguous fluorescence/lateral-flow signals and insufficient specimen volume should follow predefined repeat or recollection rules

## 6 Clinical translation and application scenarios

### 6.1 Respiratory tract infection and ICU pneumonia

Respiratory specimens are the most common clinical matrices used to evaluate CRISPR/Cas detection of Kp. Organism burdens in sputum and bronchoalveolar lavage fluid (BALF) may be higher than those in blood, but viscosity, host nucleic acid background, and amplification inhibitors can compromise assay stability. Several Kp CRISPR/Cas platforms have been preliminarily evaluated using sputum or BALF (Zhou et al., 2025; Fu et al., 2025; Pan et al., 2025; Qiu et al., 2022; Wu et al., 2025; Tan et al., 2024b). Although these studies report short amplification and Cas-reaction times, sample processing remains a major bottleneck, and a reduction in total specimen-to-report turnaround relative to existing workflows has not been demonstrated consistently. Fu et al. (Fu et al., 2025) evaluated an extraction-free approach, whereas Wu et al. (Wu et al., 2025) compared sputum-release reagents, illustrating how lysis methods directly influence amplification and Cas signals. The AMIC system developed by Xiang et al. (Xiang et al., 2024) further shows that respiratory testing requires end-to-end optimization of sample processing, amplification, readout, and reporting rather than optimization of the Cas reaction alone.

In ICU pneumonia, Kp-associated target detection alone is insufficient to guide treatment. Selected patients with severe disease, a compatible invasive syndrome, or high local resistance risk may benefit from evaluation of gene-associated resistance risk and, where clinically justified, hypervirulence-associated markers. Future respiratory panels may therefore evaluate combinations of organism-context targets, core carbapenemase genes, and selected hypervirulence-associated markers rather than report only “*K. pneumoniae* nucleic acid detected.” Mixed-specimen results should be interpreted according to the evidence levels defined in Section 5.4.

### 6.2 Bloodstream infection, sterile body fluids, and low-bacterial-load samples

Bloodstream infections and infections of sterile body fluids are clinically important but technically challenging scenarios for CRISPR/Cas platforms. Direct testing of blood or serum must address low organism burden, high host nucleic acid background, inhibitor interference, and positivity thresholds that control false-positive results. The TCC CasΦ study reported an analytical workflow of approximately 40 min after enrichment and heat lysis, demonstrating the potential of amplification-free cascade signal generation; this duration should not be interpreted as an end-to-end specimen-to-report time for the diagnosis of Kp bloodstream infection (Chen et al., 2025). The CNR platform exploited high-copy rRNA together with CuO-nanocatalytic signal amplification. After the reported 10-fold centrifugation enrichment step, the effective organism-level LOD in the original sterile-body-fluid matrix was approximately 0.69 CFU/mL. Among 64 clinical samples, the assay yielded 13 positive results, including one positive result from a culture-negative specimen that was supported by clinical follow-up and PCR. These data are therefore interpreted within the study-specific adjudication framework rather than described simply as “high agreement” with culture (Xiao et al., 2025).

These studies provide transferable engineering evidence for bloodstream-infection workflows but should not be equated with validated Kp bloodstream-infection diagnostics. Direct blood testing, positive blood culture bottle testing, and cultured-isolate testing must be evaluated separately. Direct blood testing offers the greatest potential reduction in turnaround time but is technically the most demanding. Positive blood culture bottles generally provide higher organism burdens than direct blood, whereas isolate testing is more suitable for confirmation than for early intervention. Future studies should report specimen context, processing time, reaction time, readout time, and total specimen-to-report time separately rather than group these evaluations under the broad label “clinical samples.”

Positive blood culture bottles may be a practical early entry point for prospective evaluation of CRISPR/Cas platforms. Compared with direct blood, they typically contain higher organism burdens and may be less affected by matrix inhibition after culture positivity, although performance will depend on bottle type, culture medium, organism composition, and sample processing. Compared with isolate testing, they may provide resistance-associated risk information before completion of final identification and AST. Future studies should quantify the time benefit, contamination risk, and integration of these workflows with routine MALDI-TOF MS and AST.

### 6.3 Urinary tract infection, phenotypic AST, and antimicrobial stewardship

Urinary tract infection provides a useful model for integrating CRISPR/Cas into antimicrobial stewardship. TubeCARE was designed as a prototype near-patient workflow that integrates urine processing and *blaKPC* detection within a disposable tube (Yang et al., 2025). Zheng et al. (Zheng et al., 2025) evaluated asymmetric PCR (aPCR)–Cas12a in 86 clinical urine samples (Figure 6A). Seventy-seven samples were positive in the initial broad bacterial screen, and 45 contained one of the six organisms included in the species-identification panel; the species calls for these panel-covered specimens were concordant with culture/MALDI-TOF MS. The complete reported identification-plus-AST workflow required approximately 5.5 h, including the antibiotic-exposure step, and should therefore be distinguished from the shorter times reported for individual molecular-reaction steps. Hu et al. (Hu et al., 2025) developed SEE-phAST, which uses digital RAA–CRISPR and spatially encapsulated emulsions to quantify genomic DNA changes after short antibiotic exposure. The reported Kp–ceftazidime demonstration was performed in an artificial urinary-tract-infection model rather than a prospective clinical cohort; clinical categorical-AST validation therefore remains necessary.

These studies show that CRISPR/Cas can be extended from detecting the presence or absence of resistance genes to measuring phenotypic responses. For Kp infections, genotypic testing can provide an early resistance-risk signal, whereas CRISPR-assisted rapid AST more directly addresses antimicrobial selection. Under defined specimen and platform conditions, the two approaches could form a stratified stewardship pathway: an early resistance gene warning followed by phenotypic response assessment. Prospective studies are required to demonstrate a reduction in time to an actionable result and effects on treatment decisions. Evaluation should follow diagnostic-stewardship principles that link test ordering, result interpretation, and downstream actions (Fabre et al., 2023).

### 6.4 Infection prevention and control, active screening, and one health surveillance

The risk of in-hospital transmission of CRKP isolates, particularly those showing a suspected resistance–hypervirulence convergence pattern, supports the evaluation of active-screening applications. Potential settings include rectal colonization screening, admission screening in high-risk units, and environmental monitoring. Hyeon et al. (Hyeon et al., 2026) evaluated one-pot RPA–Cas12a detection of *blaKPC* and *blaNDM* in rectal swabs, supporting its potential use for CRE colonization screening. Ji et al. (Ji et al., 2026) extended Kp and *blaOXA-48* detection to clinical, food, and environmental samples, providing One Health-oriented surveillance evidence rather than direct validation of a universal clinical Kp panel. Related ARG studies provide additional transferable evidence within a One Health framework (Vargas-Reyes et al., 2026; Wang et al., 2024).

In One Health settings, selected CRISPR/Cas implementations may offer portability, rapid target reconfiguration, and minimal-instrumentation readout. Field surveillance should nevertheless be evaluated in terms of sample processing, reagent stability, cost, quality control performance, and the capacity for source tracing rather than solely on the basis of a low LOD. CRISPR/Cas may serve as a screening tool, but positive results require culture recovery and, when transmission or source attribution is under investigation, AST and genomic confirmation.

### 6.5 Integration with WGS, mNGS, and clinical reporting systems

CRISPR/Cas, AST, WGS, and mNGS should form a complementary diagnostic continuum. CRISPR/Cas provides early target-based screening, AST confirms phenotypic resistance, WGS resolves genetic background, and mNGS supports complex-sample and unknown-pathogen identification. For suspected antimicrobial resistance–hypervirulence convergence risk signals, a CRISPR/Cas report may warrant isolate preservation, infection control risk assessment, and consideration of WGS-based confirmation. However, it should not replace genomic evidence of same-strain carriage or plasmid-level colocalization.

Clinical reports should avoid listing isolated results such as “*blaKPC* positive” or “*rmpA* positive” without organism and specimen context. A more informative report should state the validated Kp-associated or KpSC-associated organism context, list each resistance-associated and hypervirulence-associated target, describe any specimen-level convergence-risk pattern, and state the relevant limitations. It may also identify circumstances in which AST, isolate preservation, infection control review, or WGS confirmation should be considered within a locally validated pathway. This format translates molecular findings into bounded prompts for confirmatory testing and clinical review without presenting the panel as an independent diagnosis.

From a translational perspective, we propose positive blood culture bottles, rectal swab colonization screening, and high-burden respiratory specimens as plausible early entry points for prospective evaluation of Kp CRISPR/Cas risk panels. These scenarios combine comparatively high target burdens or established screening workflows with potential links to infection control review, empirical-therapy reassessment, or WGS prioritization. Direct blood and amplification-free serum testing may offer the greatest reduction in turnaround time but remain constrained by low organism burden, host nucleic acid background, threshold selection, and false-positive control; they are therefore priorities for further technical and clinical validation. Urine is suitable for evaluating stewardship pathways that combine molecular screening with rapid phenotypic AST. Table 4 compares these proposed entry scenarios and their principal validation requirements.

**TABLE 4:** Candidate clinical entry scenarios for prospective evaluation of Kp CRISPR/Cas risk panels.

Scenario	Rationale for prospective evaluation	Suggested panel scope	Critical validation issues	Potential action link after local validation
High-burden respiratory specimens and ICU pneumonia	Most Kp studies have used sputum or BALF; organism burden may permit rapid detection, and results may support early therapy review or infection control assessment	Kp/KpSC-associated target(s) plus core carbapenemases; add candidate priority hypervirulence-associated markers in severe disease or compatible clinical syndromes	Viscosity, inhibitors, specimen quality, colonization versus infection, mixed pathogens, and extraction/lysis stability	Early risk report linked to culture/AST and local isolation policy
Positive blood culture bottles	Higher organism burden than in direct blood and a plausible time-saving point before final identification and AST	Kp/KpSC-associated target(s) plus core carbapenemases and selected hypervirulence-associated markers	Bottle type, polymicrobial growth, time from positivity, contamination, and integration with MALDI-TOF/AST.	Consider accelerated AST, isolation assessment, and WGS prioritization according to validated local workflows
Direct blood or sterile body fluid	Greatest potential reduction in turnaround time for invasive infection	Focused Kp/KpSC-associated target(s) and a high-priority resistance panel; amplification-free/high-copy routes are investigational	Very low burden, host nucleic acid background, inhibitors, positivity-threshold selection, false-positive risk, enrichment time, and reference-method adjudication	Research/validation pathway; seek timely confirmation by culture and orthogonal molecular testing
Urine and urinary tract infection	Accessible specimen type for evaluating integration of genotypic warning with rapid phenotypic AST	Kp/KpSC-associated target(s) plus *blaKPC* or other locally relevant resistance genes; optional CRISPR-assisted rapid AST.	Bacterial-count thresholds, mixed flora, prior antibiotics, drug-exposure protocol, and categorical AST agreement	Antimicrobial-stewardship support and earlier targeted therapy
Rectal or admission colonization screening	Direct link to infection prevention actions and demonstrated feasibility for *blaKPC*/*blaNDM* screening	Core carbapenemase panel; a Kp/KpSC-associated target is optional depending on whether the program screens CRE or Kp specifically	Colonization prevalence, enrichment, turnaround-time benefit, positive predictive value, and isolation/de-isolation policy	Cohorting, contact precautions, and outbreak prevention according to local policy
Outbreak, environmental, food, or One Health surveillance	Portable and reprogrammable testing may broaden rapid screening beyond the clinical laboratory	Kp/KpSC-associated target(s) plus locally relevant resistance genes; add clonal/mobile-element or virulence markers when the surveillance question requires them	Sampling design, reagent stability, field controls, source attribution, and confirmatory isolate/WGS capacity	Screening and prioritization for culture, AST, and genomic source tracing

Across these clinical scenarios, the value of CRISPR/Cas depends on the complete specimen-to-report pathway rather than on the Cas reaction alone. Respiratory, blood, urine, rectal-screening, and surveillance applications differ in organism burden, preprocessing requirements, prevalence, and the action triggered by the result. A clinically interpretable workflow should therefore define the intended specimen, target combination, report category, invalid/discordant rules, and downstream AST or genomic confirmation before prospective evaluation.

## 7 Discussion: challenges and future directions

### 7.1 Sample pretreatment as a key bottleneck in clinical translation

Sample preparation is an integral component of the diagnostic platform rather than a secondary preanalytical step. Respiratory specimens can contain relatively high organism burdens but are affected by viscosity, mucus, host DNA, and amplification inhibitors. Direct blood and sterile body fluids may contain very low bacterial burdens together with abundant host nucleic acids and therefore may require enrichment, host-background management, or highly efficient extraction. Urine is comparatively accessible but varies in bacterial count, pH, salts, prior antibiotic exposure, and mixed-flora content. Rectal swabs are appropriate for colonization screening but contain complex microbial backgrounds and do not indicate infection. Positive blood culture bottles provide higher organism burdens after culture positivity but introduce broth- and bottle-specific matrices. Matrix-specific nuclease activity and nucleic-acid degradation during collection, transport, or processing can further reduce recoverable target molecules. Across all specimen types, lysis, extraction recovery, inhibitor removal, enrichment, input volume, and sample fraction entering the final reaction should be validated as explicit parts of the assay rather than treated as fixed upstream assumptions.

### 7.2 Sensitivity, specificity, contamination control, and quantitative interpretation

CRISPR/Cas studies often report very low LODs; however, the input matrix, sample-processing workflow, reference method, and threshold-setting procedure must be clearly specified. Low LODs in amplification-assisted platforms are often accompanied by a risk of aerosol contamination. Target-amplification-free platforms reduce amplification carryover but may rely on high nucleic acid input, nanomaterials, or signal cascades. Specificity cannot be established using only a small set of non-target bacteria; evaluation should include closely related KpSC species, homologous resistance gene variants, and complex clinical matrices.

Quantitative interpretation also remains challenging. Cas collateral cleavage is a nonlinear enzymatic signal amplification process, and endpoint fluorescence is not necessarily linearly correlated with target copy number. Cascade signal amplification methods such as TCC and CNR can improve sensitivity but may narrow the linear range or complicate background threshold setting. Clinical reports should therefore use qualitative outputs or prospectively validated semiquantitative signal categories rather than overstate quantitative precision.

### 7.3 Genotype–phenotype discordance and differentiation between infection and colonization

Detection of a resistance gene is not equivalent to phenotypic resistance, and detection of a virulence gene is not equivalent to a hypervirulent phenotype. Gene expression, regulatory mutations, copy-number changes, and host factors can alter genotype–phenotype relationships. As defined in Section 5.4, CRISPR/Cas results should therefore be interpreted as bounded risk indications.

In non-sterile specimens, nucleic acid positivity does not by itself distinguish infection from colonization, carriage, contamination, or residual DNA. This is particularly relevant to sputum, endotracheal aspirates, rectal swabs, and urine, where Kp may be present without invasive disease. Interpretation should therefore incorporate specimen quality, intended use, organism burden or prospectively validated semiquantitative categories, culture findings, host clinical features, and—when the test is used for screening rather than diagnosis—the prevalence of colonization. The general genotype–phenotype and co-detection boundaries are defined in Section 5.4 and are not repeated here.

### 7.4 Standardized validation and regulatory translation

Future CRISPR/Cas validation should be organized into explicit domains: (i) intended use and target definition; (ii) *in silico* and wet-lab analytical specificity/inclusivity; (iii) analytical sensitivity and LOD under defined matrix and input conditions; (iv) precision and reproducibility; (v) interference and cross-reactivity; (vi) robustness, stability, and contamination control; (vii) specimen adequacy and internal/process controls; (viii) multiplex competition and target dropout; (ix) reference methods and clinical cohort design; (x) diagnostic sensitivity and specificity; (xi) predictive values or likelihood ratios in the intended prevalence setting; and (xii) clinical or workflow utility. Analytical sensitivity should not be used as a synonym for diagnostic sensitivity, and the ambiguous term “clinical sensitivity” should be avoided unless explicitly defined.

For terminology, LOD refers to the lowest analyte level meeting a predefined detection criterion under specified analytical conditions; analytical sensitivity describes detection performance under controlled analytical conditions; and diagnostic sensitivity is the proportion of reference-positive specimens correctly identified in a defined clinical cohort. The term “clinical sensitivity” should be avoided unless it is explicitly defined.

A clinically deployable assay should predefine how specimen-adequacy controls, extraction/process controls, amplification controls, Cas/reporter-activity controls, positive controls, no-template controls, and inhibition controls are interpreted. Failure of a required control should produce an invalid result rather than a negative result. The validation protocol should also define insufficient-volume, ambiguous-signal, discordant-target, repeat-testing, and recollection rules before clinical evaluation.

Sensitivity and specificity alone are insufficient to establish utility because predictive values depend on prevalence. Intended-use studies should therefore report prevalence together with PPV/NPV and, where appropriate, likelihood ratios, particularly for low-prevalence colonization screening and hypervirulence-associated markers. Clinical validation should preferably be conducted in prospective, multicenter cohorts using consecutively collected specimens and should evaluate invalid-test rates, total time to a reportable result, reference-method agreement, and effects on treatment, infection control, WGS prioritization, or outbreak workflows. A minimum analytical, clinical, and reporting checklist is provided in Supplementary Table S4.

### 7.5 Qualitative comparison with existing diagnostic pathways

The clinical value of CRISPR/Cas platforms depends on whether they provide incremental benefit within existing diagnostic pathways. Compared with culture and AST, CRISPR/Cas can provide earlier target-level risk information in selected workflows but cannot generate a complete phenotypic susceptibility profile. Compared with qPCR, some CRISPR/Cas implementations may require less instrumentation, use isothermal reactions, or support portable readouts; qPCR remains more mature in automation, quantitative stability, quality control, and clinical laboratory standardization. CRISPR/Cas is therefore better positioned as a focused screening or complementary near-patient tool in selected scenarios than as a general replacement for qPCR. Compared with WGS or mNGS, selected CRISPR/Cas implementations may reduce instrumentation requirements, per-test complexity, or target-to-result time, but these advantages have not been established uniformly and should be evaluated using complete specimen-to-report workflows and local cost models. The most defensible role of CRISPR/Cas is to provide an early, bounded signal that may support antimicrobial stewardship review, infection control assessment, and confirmatory testing.

A shorter analytical reaction time or a lower LOD should not be presented as evidence of clinical benefit unless the study measured an intended-use workflow endpoint. In this review, “demonstrated clinical or workflow utility” is reserved for outcomes actually evaluated in clinical specimens or implementation studies; proposed effects on stewardship, isolation, WGS prioritization, or patient management are described as future or potential utility unless prospectively measured.

Economic evaluation should likewise be performed at the pathway level rather than inferred from reaction simplicity. Relevant components include per-test enzymes and reagents, extraction or cartridge consumables, device and reader costs, technician hands-on time, quality control materials, repeat or invalid-test costs, maintenance, and downstream culture, AST, or genomic confirmation. A low-cost Cas reaction may not produce a low-cost diagnostic pathway if substantial upstream processing or confirmatory testing remains necessary. Comparative cost-effectiveness therefore requires intended-use studies that measure the complete diagnostic pathway and the clinical actions triggered by the result.

### 7.6 Toward sample-to-answer and deployable platforms

Future development should shift from reaction-level sensitivity toward sample-to-answer reliability. A deployable platform should integrate closed sample processing, stable reagents, controlled reaction timing, automated readout, and result interpretation software. Near-patient or field claims additionally require validation across realistic temperature and humidity ranges, freeze-thaw exposure, lyophilization and rehydration, shelf life, shipping and transport conditions, operator variability, device-to-device variability, cartridge/material consistency, and lot-to-lot Cas/reagent activity. Microfluidics, digital droplets, and smartphone-, electrochemical-, or nanomaterial-based readouts provide candidate engineering routes, but increasing device sophistication shifts the validation burden from the Cas reaction to the complete chain of sample handling, surface chemistry, reagent stability, signal acquisition, software interpretation, and inter-device consistency. Deployable systems should therefore demonstrate robust specimen-to-report performance and measurable workflow benefit outside tightly controlled laboratory conditions rather than merely report a low analytical LOD.

For Kp, we propose prioritizing scenarios with a plausible link to clinical or infection control decisions: risk panels for high-burden respiratory specimens, rapid identification and AST in urine, early warning from positive blood culture bottles, and Kp/CRE colonization screening for locally prevalent carbapenemase markers. Prioritization should consider sample-processing difficulty, total time to a reportable result, potential effects on treatment or infection control workflows, and integration with subsequent AST or WGS confirmation.

Although the present review focuses on diagnostics, rapid molecular resistance profiling may also complement emerging pathogen-specific therapeutic research by defining resistance-associated context earlier in the clinical pathway. For example, computational work has identified the conserved zinc-dependent metallohydrolase DapE as a potential *K. pneumoniae* antimicrobial target and proposed candidate zinc-binding inhibitors using virtual screening, molecular docking, molecular dynamics, and MM/PBSA analysis (Biswas and Anbarasu, 2025). Such studies remain preclinical and do not establish treatment efficacy; CRISPR/Cas detection would therefore provide molecular context rather than select or validate a DapE-directed therapy. This diagnostic–therapeutic interface should be evaluated only as pathogen-specific therapeutic strategies progress through experimental validation.

### 7.7 Limitations of this review

This review is a structured narrative synthesis rather than a formal systematic review or meta-analysis. The available studies are highly heterogeneous in target selection, assay architecture, specimen matrix, sample-processing workflow, input volume, analytical units, and reference methods, precluding direct quantitative pooling. Many studies rely on cultured isolates, spiked matrices, or small single-center specimen sets, and few evaluate clinical outcomes or decision-making impact. Selective publication and preferential reporting of favorable analytical results may also affect the apparent maturity of the evidence base. The proposed target panel and reporting framework are based on published evidence and biological plausibility but have not been prospectively calibrated or externally validated. Rapid developments in CRISPR diagnostics and Kp epidemiology may affect the completeness of the evidence summarized here.

## 8 Conclusion

CRISPR/Cas diagnostics provide a programmable route for detecting Kp/KpSC-associated organism context, antimicrobial resistance-associated genes, and hypervirulence-associated markers, with the strongest Kp-focused evidence currently concentrated in Cas12a/Cas12b workflows for organism-context and core resistance gene detection. The literature also demonstrates important engineering progress in one-pot reactions, portable readouts, multiplexing, and CRISPR-assisted phenotypic AST, but most studies remain limited by heterogeneous matrices, small or selected cohorts, isolate/spiked-sample designs, and incomplete demonstration of clinical or workflow benefit.

The central contribution of this review is therefore not a claim that CRISPR/Cas can independently diagnose CRKP, hvKp, or resistance–hypervirulence convergence, but a conceptual framework for clinically interpretable risk reporting. The proposed four-layer target-to-report framework links validated organism context, resistance-associated targets, hypervirulence-associated targets, and contextual background to bounded report categories and predefined confirmation pathways. It explicitly separates genotype from phenotype, colonization from infection, and specimen-level co-detection from same-strain or plasmid-level convergence. CRISPR/Cas is accordingly positioned as a complementary early screening layer that may prompt AST, isolate preservation, infection control assessment, or genomic confirmation.

Future studies should prospectively evaluate target inclusivity and specificity across genomic diversity, diagnostic accuracy across specimen types and prevalence settings, complete specimen-to-report turnaround, invalid/repeat rates, antimicrobial stewardship and infection-control impact, pathway-level cost, and the frequency and consequences of molecular misclassification. Prospective multicenter studies should determine whether predefined target combinations and complete sample-to-report workflows reduce the time to actionable decisions while preserving the distinction between genotype and phenotype, colonization and infection, and specimen-level co-detection and same-strain carriage. Multicenter external validation is required before the proposed reporting categories can be considered clinical decision rules or broadly deployable diagnostic standards.
