# Supplementary material for: CRISPR/Cas diagnostics for Klebsiella pneumoniae: from resistance and hypervirulence markers to clinically interpretable risk reporting
Source: Front Bioeng Biotechnol. 2026 Sep 11;14:1949504. doi: 10.3389/fbioe.2026.1949504 (PMC13613015; doi:10.3389/fbioe.2026.1949504)
Supplement: Supplementary file 1 [file DataSheet1.pdf]

Supplementary Material

1 Supplementary Note 1. Operational definitions and interpretive boundaries

Kp versus KpSC detection. A result should be described as *K. pneumoniae* sensu stricto only when analytical validation demonstrates discrimination from closely related members of the *K. pneumoniae* species complex. Otherwise, “KpSC-associated target detected” is the more defensible wording.

Carbapenemase-associated resistance risk. Detection of *blaKPC*, *blaNDM*, *blaOXA-48-like*, *blaIMP*, *blaVIM*, or another validated carbapenemase target indicates a molecular resistance-risk signal. It does not by itself establish a phenotypically confirmed CRKP classification. In mixed or non-isolate specimens, co-detection of a Kp-associated or KpSC-associated target and a carbapenemase marker does not establish that the marker is carried by Kp.

Hypervirulence-associated risk. Candidate priority markers include aerobactin loci (*iucABCD*/*iutA*) and the virulence-plasmid-associated biomarker *peg-344*, with *rmpA*/*rmpA2* detection and a compatible clinical syndrome providing supporting context. Short-target detection does not establish full-locus integrity, absence of truncation, or expression. Integrity may be assessed by multi-region amplification, targeted sequencing, WGS, or long-read sequencing, as appropriate to the claim. No single gene, capsular type, sequence type, *magA* result, or string test independently defines hvKp. The proposed marker prioritization is conceptual and requires prospective validation.

Suspected resistance–hypervirulence convergence. Concurrent detection of Kp-associated or KpSC-associated organism-context targets, resistance-associated targets, and candidate priority hypervirulence-associated targets may prompt a predefined confirmatory workflow. In primary or mixed specimens, this pattern represents specimen-level co-detection and cannot prove that the targets are carried by the same strain or genetic vehicle.

Evidence-level terminology. “Specimen-level co-detection” indicates concurrent target detection without organism linkage. “Probable same-organism association” may be used only when independent evidence strongly supports a dominant or apparently monomicrobial Kp context but same-strain genomic confirmation is absent. “Confirmed same-strain resistance–hypervirulence convergence” requires a recovered isolate/single-colony lineage with appropriate evidence of organism identity, resistance, hypervirulence-associated markers, and genomic linkage; plasmid-level colocalization requires plasmid-resolved evidence.

Negative panel interpretation. “Not detected” applies only to the targets included in the validated panel and within its established analytical limits. It cannot exclude untested resistance mechanisms, regulatory mutations, variant-associated target dropout, low organism burden, or resistance genes carried by organisms outside the validated organism context. A negative result should not be reported when a required specimen-adequacy, extraction/process, amplification, Cas/reporter, or inhibition control has failed.

Colonization versus infection. Nucleic-acid detection in sputum, urine, rectal swabs, or other non-sterile specimens must be interpreted with specimen quality, a validated organism-burden estimate when available, culture findings, and the clinical syndrome. The molecular panel is a risk-stratification layer, not an independent diagnosis of active infection.

2 Supplementary Note 2. Proposed structure for standardized result reports

1) Validity statement: report specimen and process-control status and whether predefined validity criteria and thresholds were met.

2) Organism context: state the exact validated claim, such as “Kp-associated nucleic acid detected” or “KpSC-associated target detected,” and avoid stronger species attribution than the analytical validation supports.

3) Target list: report each resistance-associated and hypervirulence-associated target as detected, not detected, or indeterminate rather than presenting only a composite label.

4) Risk interpretation: translate the combination into a bounded category—organism-context detection only, gene-associated resistance risk, hypervirulence-associated risk, or suspected resistance–hypervirulence convergence risk.

5) Limitations: state that genotype does not replace phenotypic AST, a virulence-associated marker does not independently establish hvKp, and specimen-level co-detection does not prove organism linkage, same-strain carriage, or genetic colocalization.

6) Action prompt: indicate when culture or isolate recovery, AST, isolate preservation, infection control assessment, targeted sequencing, WGS, or mNGS may be considered according to the result, specimen context, and local workflow.

7) Invalid or discordant results: predefine repeat testing, recollection, orthogonal testing, and escalation rules for control failure, organism-context-negative/ARG-positive patterns, or results that conflict with culture or AST.

Example high-priority report

Result: A Kp-associated or KpSC-associated target, a carbapenemase marker, and one or more candidate priority hypervirulence-associated markers were detected in this specimen.
Interpretation: This pattern represents a suspected resistance–hypervirulence convergence risk signal.
Limitation: Specimen-level co-detection does not establish that the resistance marker is carried by Kp or that all detected targets are present in the same strain or genetic vehicle.
Potential confirmatory actions: Isolate recovery, phenotypic AST, infection control risk assessment, isolate preservation, and WGS or plasmid-level analysis may be considered according to the clinical context and local workflow.

Supplementary Table S1. Literature-search strategy used for this structured narrative review.

Source/database	Search purpose	Exact search string / source-specific query	Search/update date	Notes
PubMed/PMC	Broad Kp/CRISPR diagnostic search	("Klebsiella pneumoniae"[Title/Abstract] OR "K. pneumoniae"[Title/Abstract] OR CRKP[Title/Abstract]) AND (CRISPR[Title/Abstract] OR "CRISPR-Cas"[Title/Abstract] OR Cas12a[Title/Abstract] OR Cas12b[Title/Abstract] OR Cas13a[Title/Abstract] OR CasΦ[Title/Abstract] OR CasPhi[Title/Abstract] OR dCas9[Title/Abstract]) AND (detect*[Title/Abstract] OR diagnos*[Title/Abstract] OR assay[Title/Abstract] OR biosensor[Title/Abstract])	6 July 2026	Principal bibliographic entry search.
PubMed/PMC	Direct Kp detection and one-pot/extraction-free platforms	("Klebsiella pneumoniae"[Title/Abstract] OR "K. pneumoniae"[Title/Abstract]) AND (Cas12a[Title/Abstract] OR Cas12b[Title/Abstract] OR Cas13a[Title/Abstract] OR CRISPR[Title/Abstract]) AND (RPA OR RAA OR LAMP OR ERA OR "one-pot" OR "one-tube" OR "extraction-free")	6 July 2026	Targeted supplementation for direct Kp assay studies.
PubMed/PMC	CRKP/carbapenemase-associated targets	(CRKP[Title/Abstract] OR "carbapenem-resistant Klebsiella pneumoniae"[Title/Abstract] OR CRE[Title/Abstract] OR Enterobacterales[Title/Abstract]) AND (CRISPR OR Cas12a OR Cas13a OR Cas12b) AND (blaKPC OR KPC OR blaNDM OR NDM OR "OXA-48" OR GES OR carbapenemase)	6 July 2026	Targeted resistance gene and carbapenemase search.
PubMed/PMC	hvKp/hypervirulence-associated targets	("hypervirulent Klebsiella pneumoniae" OR hvKp OR hvKP) AND (CRISPR OR Cas12a OR Cas13a OR CasPhi OR CasΦ) AND (rmpA OR rmpA2 OR iucA OR iroB OR "peg-344" OR virulence)	6 July 2026	Targeted hypervirulence marker search.
PubMed/PMC	Multiplex, readout, and scenario-specific platforms	("Klebsiella pneumoniae" OR CRKP OR respiratory OR urine OR sputum OR "blood culture" OR "food safety") AND (CRISPR OR Cas12a OR Cas13a OR Cas12b OR dCas9) AND (multiplex OR simultaneous OR microfluidic OR smartphone OR electrochemical OR SERS OR "lateral flow")	6 July 2026	Targeted search for multiplexing, POCT readouts, and clinical/field scenarios.
ScienceDirect/Elsevier	Publisher-platform supplementation	"Klebsiella pneumoniae" CRISPR Cas12a; "Klebsiella pneumoniae" CRISPR Cas13a; CRKP CRISPR; blaKPC CRISPR Klebsiella	6 July 2026	Used to supplement Elsevier-hosted studies and recent/online-first records.
Frontiers	Direct Kp, KPC, and CRKP assay studies	"Klebsiella pneumoniae" "CRISPR/Cas12a"; "Klebsiella pneumoniae" "KPC resistance gene"; "visual detection platform" "CRKP"	6 July 2026	Targeted publisher-site supplementation.
ASM Journals	Kp detection and microbiology-platform studies	"Klebsiella pneumoniae" CRISPR; "Klebsiella pneumoniae" Cas12b; "Rapid and simultaneous detection" Klebsiella pneumoniae	6 July 2026	Targeted publisher-site supplementation, including Microbiology Spectrum.
ACS Publications	Materials, devices, multiplexing, and portable readouts	"Klebsiella pneumoniae" CRISPR; "kpc-2" CRISPR polymer dot; "Klebsiella" SERS CRISPR; "hvKp" CRISPR smartphone	6 July 2026	Targeted supplementation across ACS diagnostic and biosensor journals.
Springer / MDPI / DovePress / RSC / Wiley	Resistance-gene and method-specific supplementation	"blaKPC" CRISPR Cas13a; "blaKPC" Cas12a Klebsiella; "Klebsiella pneumoniae" "Analytical Methods" CRISPR	6 July 2026	Supplementary publisher-site searches for resistance and method studies.
SinoMed / Chinese-language databases and journal websites	Chinese-language Kp/CRISPR studies	肺炎克雷伯菌 AND (CRISPR OR Cas12a OR Cas13a OR Cas12b OR CasΦ) AND (检测 OR 诊断 OR 快速检测 OR RPA OR RAA OR ERA OR LAMP)	6 July 2026	Supplementary retrieval of Chinese-language original/technical-method studies.

Notes: The table lists source-specific searches documented as completed in the authors' search log on 6 July 2026. No lower publication-date limit was applied; the documented searches covered records available from database inception through 6 July 2026. PubMed/PMC served as the principal bibliographic entry point, with publisher-platform and Chinese-language searches used for supplementary coverage. Search terms were adapted iteratively to organism-context detection, carbapenemase and other resistance-associated targets, hypervirulence-associated markers, multiplex/readout strategies, specimen-specific workflows, and rapid antimicrobial susceptibility testing. Study eligibility, exclusion criteria, deduplication, and screening procedures are described in Section 1.1 of the revised manuscript.

Abbreviations: ACS, American Chemical Society; ASM, American Society for Microbiology; CRKP, carbapenem-resistant Klebsiella pneumoniae; CRE, carbapenem-resistant Enterobacterales; hvKp, hypervirulent K. pneumoniae; Kp, K. pneumoniae; LAMP, loop-mediated isothermal amplification; LFA, lateral-flow assay; PMC, PubMed Central; POCT, point-of-care testing; RAA, recombinase-aided amplification; RPA, recombinase polymerase amplification; RSC, Royal Society of Chemistry; SERS, surface-enhanced Raman scattering.

Supplementary Table S2. Study-level evidence inventory for CRISPR/Cas-based Kp organism-context, resistance gene, hypervirulence marker, and rapid-AST applications discussed in the review.

Study/platform	Target(s)	Assay/workflow	Readout	Validation/sample context and evidence context	Evidence contribution and major limitation
Qiu et al., CRISPR-top (74)	Kp *rcsA*	One-pot LAMP-Cas12b with PAM introduced through primers	Fluorescence	64 Kp strains, 41 non-Kp strains; 58 sputum samples [strain panel + primary sputum]	Demonstrated secondary CRISPR recognition after LAMP; 96% sensitivity and 100% specificity versus culture in the reported cohort. LOD about 1 pg DNA/reaction; focused on species identification.
Tan et al. (119)	Kp *rcsA*	RPA–Cas12a	Fluorescence and LFA	25 clinical sputum samples [primary sputum; small cohort]	Rapid dual-readout respiratory screening; limited sample size and primarily species-level output.
Wu et al. (75)	Kp species target	One-tube RPA–Cas12a with physical separation of stages	Fluorescence	23 sputum samples [primary sputum; small cohort]	Reduced contamination risk from opening reaction tubes; signal after 15-min RPA plus 15-min Cas reaction. Sample-processing dependence remains.
Fu et al., EXORCA (58)	Kp species target	Extraction-free one-pot RPA–Cas12a using rapid lysis and suboptimal PAM	Fluorescence	Cultured Kp and 20 clinical samples [cultured organisms + clinical cohort]	LOD 10 CFU/μL in cultured Kp; advances closed-tube POCT but does not include resistance/virulence targets.
Pan et al. (61)	Kp rcsA	Light-controlled one-pot RPA–Cas12a with NPOM-caged crRNA	Fluorescence / UV-assisted endpoint	30 clinical Kp isolates and 20 clinical control isolates; PCR comparator [clinical isolate-based]	LOD 4.072 × 10^2 copies/reaction; reported 100% sensitivity and 100% specificity in the 50-isolate set. Temporal control reduces premature Cas activation, but DNA extraction and manual UV activation remain workflow limitations; the study is isolate-based rather than direct primary-specimen validation.
Zhou et al., CRISPR-KP (43)	Kp species target	AapCas12b + engineered MCDA, one-pot at 59 °C	Fluorescence	83 BALF samples; comparisons with culture/ID, qPCR, and PCR [primary BALF cohort]	LOD 10 fg/reaction (~1.6 copies). This reaction-level value should not be interpreted as a clinically relevant BALF burden or converted to a CFU/mL threshold without validated specimen-recovery, extraction/elution, and reaction-input parameters. Discordant positives require independent adjudication; extraction and reader remain necessary.
Tan et al. (76)	12 respiratory pathogens including Kp	Paraffin-partitioned one-pot RPA–Cas12a	Visual/fluorescence platform	Respiratory multipathogen platform evaluation; Kp-specific clinical details are limited [multipathogen evaluation; Kp details limited]	Shows broad respiratory multiplex feasibility; pathogen panel does not provide Kp resistance–hypervirulence stratification.
Xiang et al., AMIC (77)	12 respiratory bacteria including Kp	On-chip extraction, lyophilized RAA–Cas13a	Automated microfluidic readout	60 clinical samples; ~40-min workflow [clinical cohort; automated platform]	Sample-to-answer engineering and harmonized reactions; equipment and cartridge complexity exceed simple bedside formats.
Xie et al., MiND-DMF (78)	Six bacterial species including Kp	Digital microfluidic multiplex nucleic acid detection with on-chip DNA extraction, RPA, and Cas12a in ~1.5-μL droplets	Chip-based fluorescence	50 multi-source clinical samples; 48 results concordant with qPCR, with two MiND-DMF-positive/qPCR-negative samples whose positive results were subsequently supported by 16S rRNA Sanger sequencing [multi-source clinical cohort]	LOD 100 CFU/mL for all six bacterial targets; reported sample-to-answer time 55 min; reported sensitivity of 100% and specificity of 98–100%. Resistance and virulence targets were not included.
Wei et al. (79)	*E. coli uidA + Kp rcsA*	Dual RPA–Cas12a	Fluorescence	30 *E. coli* isolates, 30 Kp isolates, 20 non-target isolates [isolate-based]	Useful mixed-pathogen screening concept with reported agreement with PCR in the tested isolate set; not validated as a primary-specimen risk panel.

Study/platform	Target(s)	Assay/workflow	Readout	Validation/sample context and evidence context	Evidence contribution and major limitation
Tan et al. (54)	Kp *rcsA + blaKPC*	Dual RPA with target-specific Cas12a readouts	Fluorescence	Kp and KPC assay evaluation; detailed specimen-design information could not be extracted unambiguously from the source report [design details limited]	Provides a clinically intuitive linkage between organism identity and resistance but still requires target-specific readouts and does not constitute an operationally integrated single-tube multiplex assay/report.
Curti et al. (80)	KPC, NDM, OXA-48 and other targets	RPA/RT-RPA–Cas12a	Fluorescence or LFA	Analytical proof-of-concept pathogen/ARG testing [analytical proof-of-concept]	Early demonstration of a reusable Cas12a architecture for carbapenemase genes; target-level detection requires organism context.
Xu et al. (81)	KPC and NDM	LAMP–Cas12a	Lateral-flow strip	Predominantly isolate-based validation [predominantly isolate-based]	Supports low-cost visual screening but has a restricted target spectrum and requires control of nonspecific LAMP amplification.
Li et al. (82)	*blaKPC*	Comparison of PCR-, LAMP-, and RPA–Cas12a	Fluorescence	Carbapenem-resistant Enterobacterales evaluation; detailed specimen-design information could not be extracted unambiguously from the source report [CRE evaluation; details limited]	RPA–Cas12a showed the most favorable overall performance across the evaluated metrics; LAMP showed a risk of cross-reactivity. The comparison was assay- and target-specific. Findings emphasize target-specific optimization.
Gao et al. (83)	*blaKPC in Kp*	PCR–Cas12a	Fluorescence	KPC-producing CRKP assay evaluation; detailed specimen-design information could not be extracted unambiguously from the source report [CRKP evaluation; details limited]	Cas12a provides a sequence-confirmation layer after PCR; the workflow is less operationally integrated than later closed-tube isothermal approaches.
Shin et al. (84)	*blaNDM / antimicrobial resistance genes*	PCR–Cas12a	Fluorescence	CRE and surveillance-oriented specimens; detailed specimen-design information could not be extracted unambiguously from the source report [surveillance-oriented; details limited]	Supports post-amplification specificity and SNV discrimination; not a universal replacement for amplification.
Liang et al. (85)	*blaKPC*	RPA–T7–Cas13a	Fluorescence/LFA-compatible	117 clinical strains and 311 sputum specimens [isolates + primary sputum]	The study provides a large validation set for the CRKP-associated target; the added transcription step increases workflow complexity.
Cao et al. (44)	Kp + *blaKPC* + *blaNDM*	PCR- or RAA-T7-Cas13a	Fluorescence and LFA	Clinical CRKP assay evaluation; detailed specimen-design information could not be extracted unambiguously from the source report [clinical CRKP; details limited]	The PCR-CRISPR format had an LOD of 1 copy/µL, whereas the RAA formats had LODs of about 10 copies/µL. The workflow provides organism-plus-resistance targets but retains a transcription step.
Patil et al., PathCrisp-NDM (86)	Conserved NDM regions / multiple variants	LAMP–Cas12a using crude colony or broth lysate	CRISPR endpoint readout	Cultured isolates or culture-positive material [isolate/culture-positive]	Variant-aware inclusivity and rapid culture-based confirmation; not direct primary-sample testing.

Study/platform	Target(s)	Assay/workflow	Readout	Validation/sample context and evidence context	Evidence contribution and major limitation
Xu et al. (87)	*blaKPC, blaNDM, blaOXA-48, blaIMP, blaVIM*	Multiple RPA–Cas12a	Fluorescence/visual workflow	Reported 75-item clinical evaluation; conventional PCR comparison. The source article inconsistently describes the evaluated material as clinical strains and sputum specimens, and subgroup totals are not fully internally consistent.	Targets KPC, NDM, OXA, IMP, and VIM; authors reported complete concordance with conventional PCR. Individual positive/negative subgroup counts are not reproduced because of internal source-reporting inconsistencies; the study is interpreted primarily as multi-target feasibility rather than a definitive diagnostic-performance estimate.
Ortiz-Cartagena et al. (128)	OXA-48 and GES	LAMP-T7-Cas13a	LFA	Resistant-bacteria assay evaluation; detailed specimen-design information could not be extracted unambiguously from the source report [resistant-bacteria evaluation; details limited]	Expands carbapenemase coverage beyond KPC/NDM; DNA-to-RNA conversion adds steps.
Pablo-Marcos et al. (126)	GES β-lactamase	Amplification-free Cas12a	Fluorescence	Resistance-gene detection across host and genetic contexts [analytical cross-context]	Demonstrates amplification-free feasibility but sensitivity depends on copy number, host nucleic acid background, and genetic vehicle.
Yang et al., RCCS (127)	*blaKPC and blaNDM*	RPA–Cas12a	Real-time fluorescence, endpoint fluorescence, and LFA	CRE-associated target evaluation; detailed specimen-design information could not be extracted unambiguously from the source report [CRE target evaluation; details limited]	Flexible readout; substantially different target-specific sensitivities show that panel members cannot share unverified performance claims.
Hyeon et al. (88)	*blaKPC and blaNDM*	One-pot RPA–Cas12a with modified/suboptimal PAMs	Fluorescence	44 clinical rectal swab specimens; 10-min boiling pretreatment followed by a 30-min one-pot assay; multiplex qPCR comparator [primary rectal swabs]	LOD 10^{-17} M for blaKPC and 10^{-16} M for blaNDM in the 30-min one-pot reaction; reported 100% sensitivity and 100% specificity; reported sample-to-answer time of approximately 40 min. The single-matrix cohort requires external validation.
Yang et al., TubeCARE (120)	*blaKPC*	Disposable integrated urine-processing and molecular-detection tube	POCT readout	Prototype urinary-tract-infection workflow; clinical design details are limited [prototype urine workflow; details limited]	Integrates sample processing and detection; single-target scope cannot represent all CRKP mechanisms.
Wang et al. (93)	Kp species target + *blaSHV*	PCR-LbCas12a	Fluorescence	Sputum and clinical Kp isolates [primary sputum + isolates]	Links species detection with *blaSHV*-family screening; *blaSHV*-family positivity requires allele-level and phenotypic context because not all alleles are ESBLs.
Vargas-Reyes et al., C12a toolbox (94)	blaCTX-M-15, floR, and intI1	Portable Cas12a assay	Portable fluorescence/visual workflow	Small isolate sets compared with resistance characterization, PCR/WGS, as applicable [proof-of-concept isolate evaluation]	blaCTX-M-15 detected in 7 ESBL-producing isolates and absent from 8 β-lactam-susceptible isolates; floR characterization included 11 positive and 6 negative isolates; intI1 detected in 17 of 18 ARG-positive E. coli isolates. Supports modular target-level feasibility, not broad diagnostic performance.

Study/platform	Target(s)	Assay/workflow	Readout	Validation/sample context and evidence context	Evidence contribution and major limitation
Tyumentseva et al. (95)	*blaOXA-1*	Conventional PCR preamplification followed by optimized Cas12a fluorescence detection	Fluorescence	Model-template analytical evaluation plus 50 WGS-confirmed blaOXA-1-positive and 11 negative genomic DNA samples [amplification-assisted analytical/genomic DNA evaluation]	A model-template input of 1.25 copies/reaction was detected after 65 min of PCR preamplification plus approximately 5 min of Cas12a detection; the total assay time was approximately 70 min. The near-single-copy value is amplification-assisted and is not a direct specimen-level detection threshold.
Gong et al. (101)	*mcr-1*	RPA–Cas12a	Fluorescence	Purified DNA and spiked fecal, blood, and urine matrices [spiked matrices]	The analytical LOD was 420 fg DNA. In spiked matrices, organism-level thresholds were ~6.2×10^3 CFU/mL in feces, 2.6×10^3 CFU/mL in blood, and 1.6×10^3 CFU/mL in urine (approximately 6.2, 2.6, and 1.6 CFU/reaction under the reported input conditions). Chromosomal colistin-resistance mechanisms in Kp remain outside the scope of the assay.
Wang et al. (102)	*mcr-1 + tet(X4)*	One-tube RPA–Cas12b	Fluorescence	Pork and market environmental samples [food/environmental surveillance]	The LODs were 6.25 and 9 copies, respectively; the assay has strong One Health relevance but has not been validated as a universal clinical Kp panel.
Li et al. (106)	Hypervirulence-associated genes + *tet(A)*	Violet-phosphorene-assisted RPA–Cas12a	Smartphone fluorescence imaging	Engineering proof-of-concept for on-site detection of resistant/hypervirulent Kp [engineering proof-of-concept]	The nanomaterial lowers background and supports portability; device/material reproducibility and marker weighting need clinical validation.
Bhattacharjee et al. (46)	Kp species target + *rmpA*	Paper-based RPA-Cas13a	Paper/visual readout	Hypervirulent-Kp assay study; detailed sample-design information could not be extracted unambiguously from the source report [hvKp study; details limited]	Early evidence for low-equipment screening of hypervirulence-associated risk; *rmpA* alone cannot establish hvKp and Cas13 requires transcription for DNA targets.
Huang et al. (107)	Multiple hypervirulence-associated genes	Cas12a colorimetric platform with RGB quantification	Smartphone-assisted colorimetry	Analytical evaluation of multiple virulence genes [multi-marker analytical]	Improves accessibility and provides a semiquantitative readout; target weighting and clinical syndrome linkage remain essential.
Long et al. (108)	*rmpA + peg-344*	Entropy-driven circuits regulating one Cas12a system	Multiplex fluorescence	Dual hypervirulence marker analytical evaluation [dual-marker analytical]	Addresses multiplex encoding despite shared collateral cleavage; does not independently establish a hypervirulent phenotype.
Wu et al., PEAR (114)	*khe + blaKPC-2*	PAM-engineered abasic-site probe-assisted RPA–Cas12a	Fluorescence	30 clinical sputum specimens; qPCR comparator [small single-center clinical cohort]	Analytical LOD 1 copy/µL for plasmid-derived khe and blaKPC-2 templates; reported analytical specificity of 100% in the tested panel. Reported diagnostic accuracy was 93.3% for khe and 86.7% for blaKPC-2 according to the main Results, contingency data, and Conclusion; the source abstract reports 87.7% for blaKPC-2. Artificial PAM engineering broadens target design but increases probe/enzyme optimization burden.

Study/platform	Target(s)	Assay/workflow	Readout	Validation/sample context and evidence context	Evidence contribution and major limitation
Zheng et al., aPCR–Cas12a (121)	Urinary pathogen ID + phenotypic AST response	Asymmetric PCR–Cas12a after short antibiotic exposure	Fluorescence	86 clinical urine samples; 77 positive in the initial broad bacterial screen, with 45 containing a species represented in the six-organism identification panel; culture/MALDI-TOF comparison [primary urine cohort]	Species calls for target-panel specimens were concordant with culture/MALDI-TOF MS. The complete reported ID-plus-AST workflow was approximately 5.5 h, including antibiotic exposure. The platform extends CRISPR toward phenotypic response measurement but requires drug-exposure standardization and categorical AST validation.
Hu et al., SEE-phAST (122)	Phenotypic AST; Kp–ceftazidime demonstration	Digital RAA-CRISPR in spatially encapsulated emulsions	Digital fluorescence	Kp–ceftazidime demonstration in an artificial urinary-tract-infection model [phenotypic-AST engineering model rather than prospective clinical cohort]	Demonstrates digital gDNA copy-number response measurement after antibiotic exposure; clinical categorical-AST validation in authentic cohorts remains necessary.
Ji et al. (124)	Kp + *blaOXA-48*	RPA–Cas12a	Visual readout	Clinical plus food/environmental samples [clinical + field surveillance]	Links clinical and external surveillance; positive results still require organism linkage, AST, and WGS/source-tracing confirmation.

Abbreviations: ARG, antimicrobial resistance gene; AST, antimicrobial susceptibility testing; BALF, bronchoalveolar lavage fluid; CFU, colony-forming units; CRKP, carbapenem-resistant *Klebsiella pneumoniae*; CRE, carbapenem-resistant Enterobacterales; LFA, lateral-flow assay; LOD, limit of detection; MALDI-TOF, matrix-assisted laser desorption/ionization time-of-flight mass spectrometry; MCDA, multiple cross-displacement amplification; PAM, protospacer-adjacent motif; POCT, point-of-care testing; RAA, recombinase-aided amplification; RPA, recombinase polymerase amplification; WGS, whole-genome sequencing. LOD values retain the original units and should not be compared across different matrices, inputs, or per-reaction/per-volume bases. Evidence context is descriptive, not a formal risk-of-bias score. Of the 53 retained original studies, 38 directly addressed Kp/KpSC-focused diagnostic, resistance, hypervirulence, or rapid-AST applications and are included in this inventory; the remaining retained studies provided transferable CRISPR engineering or validation evidence discussed in the main text. Where specimen- or sample-design details could not be extracted unambiguously from a source report, the inventory labels the context as "details limited" rather than inferring unsupported information.

Supplementary Table S3. Conceptual matrix of candidate target content for prospective validation across clinical and surveillance scenarios.

Clinical/surveillance scenario	Typical specimen	Candidate core identity/resistance content	Candidate optional resistance content	Candidate hypervirulence/background content	Reporting and workflow emphasis
Routine respiratory screening	High-burden sputum/BALF	Kp/KpSC-associated organism context; core local carbapenemases	ESBLs when stewardship value is established	Candidate priority hypervirulence-associated markers only when severe disease or a compatible syndrome raises the pretest probability	Do not infer infection from nucleic acid positivity alone; assess specimen quality and culture.
ICU pneumonia / severe infection	Sputum, BALF, endotracheal aspirate	Kp/KpSC-associated target(s); *blaKPC*/*blaNDM*/*blaOXA-48-like*/*blaIMP*/*blaVIM* as locally relevant	Selected last-line resistance markers	Aerobactin loci (*iucABCD*/*iutA*), *peg-344*, and *rmpA/rmpA2* targets, with locus integrity requiring sequence-level confirmation; report K1/K2 and clonal background separately as contextual information	Consider rapid reporting, AST, infection control review, and isolate preservation according to validated local workflows.
Positive blood culture bottle	Apparently monomicrobial or dominant Gram-negative growth	Kp/KpSC-associated target(s) plus core carbapenemase markers	Selected ESBL/last-line markers	Candidate priority hypervirulence-associated markers	Candidate early-entry scenario for prospective evaluation; assess polymicrobial bottles and integration with MALDI-TOF/AST.
Direct blood / sterile fluid	Low-burden invasive samples	Focused Kp/KpSC-associated target(s) plus the highest-impact resistance targets	Additional resistance targets only where analytical sensitivity has been prospectively validated.	Optional; evaluate a serial, reflex, or parallel strategy only after matrix-specific analytical validation.	Use stringent controls, enrichment/lysis validation, and orthogonal adjudication.
Urinary tract infection	Urine	Kp/KpSC-associated target(s) plus local carbapenemases	ESBL and selected urine-relevant ARGs; optional rapid phenotypic AST	Not routine unless the clinical syndrome suggests invasive hypervirulence-associated disease.	Interpret mixed flora and colony-count thresholds; link to stewardship.
Rectal/admission screening	Rectal swab	Core carbapenemases; add a Kp/KpSC-associated target if Kp-specific screening is intended	Usually not needed for first-line CRE screen	Not routine	Report colonization risk, not infection; validate enrichment and policy impact.
Outbreak investigation	Isolates, positive cultures, environmental specimens	Kp/KpSC-associated target(s) plus outbreak-relevant carbapenemases	Broader ARG set based on index strain	Virulence markers may be included; CRISPR results should trigger downstream genomic characterization rather than substitute for clonal or mobile-element analysis.	CRISPR is a screen; WGS is required for transmission and colocalization inference.
One Health surveillance	Food, animal, environmental, wastewater	Kp/KpSC-associated target(s) when source attribution is relevant	*mcr, tet(X), carbapenemases, and locally prioritized ARGs*	Optional, depending on the surveillance question.	Use culture recovery, metadata, and genomic source tracing to confirm positive screening results.

Abbreviations: ARG, antimicrobial resistance gene; AST, antimicrobial susceptibility testing; BALF, bronchoalveolar lavage fluid; CRE, carbapenem-resistant Enterobacterales; ESBL, extended-spectrum β-lactamase; ICU, intensive care unit; Kp, *Klebsiella pneumoniae*; KpSC, *K. pneumoniae* species complex; WGS, whole-genome sequencing. “Candidate core” and “candidate optional” indicate priorities for prospective evaluation, not a validated or universally recommended panel. Local prevalence, analytical performance, and the intended clinical action should determine the final target list.

Supplementary Table S4. Proposed minimum analytical, clinical, and reporting checklist for translational Kp CRISPR/Cas studies.

Validation domain	Proposed minimum reporting expectation	Why it matters
Intended use and claims	Define specimen type, operator, setting, target population, clinical question, and whether the assay is screening, confirmation, or triage.	Prevents analytical results from being generalized beyond the validated workflow.
Target definition	Provide sequence, genomic location, target function, inclusivity/exclusivity rationale, PAM/primer design, and version-controlled databases.	Essential for Kp versus KpSC claims and variant-aware ARG detection.
In silico specificity	Use large, geographically diverse, version-controlled genome collections to assess closely related KpSC species, homologous ARG families, expected variants, and relevant human/microbial background sequences.	Small convenience panels cannot establish broad specificity.
Wet-lab inclusivity/exclusivity	Use geographically and genetically diverse target strains plus clinically relevant near-neighbors and homologous genes.	Quantifies empirical cross-reactivity and variant dropout.
Sample processing	Report input volume, lysis/extraction/enrichment, inhibitor removal, nuclease exposure or nucleic-acid degradation where relevant, elution, concentration factor, and hands-on time.	The LOD of the Cas reaction alone does not represent specimen-level sensitivity.
Analytical sensitivity	Report LOD with replicate-based detection probability in each claimed matrix; distinguish copies, mass, CFU, and per-reaction versus per-mL units.	Enables interpretable comparison and guards against unit-based overstatement.
Analytical specificity/interference	Test common co-pathogens, host DNA, blood/urine/sputum interferents, antibiotics, transport media, and expected specimen contaminants.	Complex matrices can alter amplification and collateral cleavage.
Precision and robustness	Assess within-run, between-run, operator, day, device, lot, temperature, humidity, and reagent-storage effects.	Required for POCT and device-integrated deployment.
Carryover and contamination control	Document closed-tube design, physical workflow separation, negative-control frequency, and decontamination strategy.	Amplification-assisted CRISPR is vulnerable to aerosol carryover.
Controls and validity rules	Include specimen-adequacy/process control when applicable, extraction/process control, amplification control, Cas/reporter activity control, positive control, and no-template control; predefine criteria for inhibition, invalid results, repeat testing, and specimen recollection. Failure of a required internal/process control should invalidate the result rather than support a negative interpretation.	Prevents technically failed specimens from being reported as interpretable negatives or positives.
Threshold setting	Lock positivity/indeterminate thresholds before clinical evaluation; report how background and run-to-run normalization are handled.	Collateral cleavage is nonlinear and endpoint signals are not automatically quantitative.
Multiplex performance	Evaluate each target alone and in combinations across a range of concentration ratios; assess competition, dropout, cross-channel bleed, and mixed infections.	Panel-level performance cannot be inferred from singleplex assays.
Reference methods	Use culture/ID, AST, targeted PCR/sequencing, and WGS as appropriate; specify discordant-resolution procedures.	No single comparator simultaneously establishes species identity, phenotype, and colocalization.
Clinical cohorts	Prefer prospective, multicenter cohorts of consecutively collected clinical specimens; separately report isolates, positive cultures, and direct specimens.	Spiked matrices and isolate DNA do not establish clinical diagnostic accuracy.
Diagnostic accuracy	Report sensitivity, specificity, predictive values with prevalence context, confidence intervals, invalid rate, and subgroup performance.	Clinical utility depends on local pretest probability and specimen type.
Genotype–phenotype interpretation	Predefine language for gene-associated risk and negative panel scope; compare with phenotypic AST.	ARG presence is not a complete susceptibility phenotype.
Hypervirulence interpretation	Use predefined multi-marker interpretation rules; document phenotype/clinical syndrome and genomic confirmation; avoid single-marker hvKp claims.	Virulence markers differ in predictive weight and may be disrupted or unexpressed.

Validation domain	Proposed minimum reporting expectation	Why it matters
Colocalization and mixed samples	Explicitly distinguish specimen-level co-detection from organism linkage, same-strain carriage, or same-plasmid carriage; confirm by single-colony or genomic evidence.	Critical for suspected resistance–hypervirulence convergence.
Resource use and economic evaluation	Report per-test enzymes and reagents, cartridges/consumables, capital equipment and maintenance, technician hands-on time, quality-control materials, invalid/repeat-test costs, and downstream culture, AST, or genomic confirmation; compare total diagnostic-pathway resource use and cost with the current standard workflow.	Reaction-level simplicity or low reagent cost does not establish a lower total diagnostic-pathway cost or cost-effectiveness.
Turnaround and workflow	Report total and hands-on time from specimen receipt to result reporting, time associated with failures and repeat testing, and integration with routine ID/AST.	A short reaction time is not equivalent to an actionable clinical turnaround time.
Clinical impact	Measure empirical-therapy changes, time to active therapy, isolation/de-isolation timing, WGS prioritization, and outbreak workflow effects.	Analytical accuracy alone may not provide incremental clinical value.
Stability and manufacturing	Assess temperature and humidity tolerance, freeze-thaw exposure, lyophilization/rehydration, storage and shipping conditions, shelf life, cartridge/material consistency, device-to-device variation, and lot-to-lot Cas/reagent activity; define lot-release criteria and acceptance ranges.	Reagent and device stability determine deployability.
Software and connectivity	Validate algorithms, audit trails, cybersecurity, user interface, data transfer, and laboratory/hospital-system integration.	Digital readout introduces additional regulated components.
Reporting template	List organism context, individual targets, risk category, limitations, confirmation recommendations, and invalid-result language.	Transforms molecular results into cautious and actionable communication.

Abbreviations: ARG, antimicrobial resistance gene; AST, antimicrobial susceptibility testing; CFU, colony-forming units; ID, identification; Kp, *Klebsiella pneumoniae*; KpSC, *K. pneumoniae* species complex; LOD, limit of detection; PAM, protospacer-adjacent motif; POCT, point-of-care testing; WGS, whole-genome sequencing. This checklist supports study design and transparent reporting and is not a regulatory standard. It should complement, not replace, applicable STARD, MIQE, CLSI, ISO, regulatory, and local laboratory requirements.
